# Supplementary material for: Viral rhodopsins 1 are an unique family of light-gated cation channels
Source: Nat Commun. 2020 Nov 11;11:5707. doi: 10.1038/s41467-020-19457-7 (PMC7659345; doi:10.1038/s41467-020-19457-7)
Supplement: Supplementary file 1 — Supplementary Information [file 41467_2020_19457_MOESM1_ESM.pdf]

Supplementary Note 1

Unusual protein-lipid interaction in the OLPVR1 structure

As mentioned in the main manuscript, both OLPVR1 structures revealed a fragment of a lipid molecule (MP, 97N or MO, OLC) between the TM6 and TM7 helices, immersed into a pore on the intracellular side of the protein and also a groove at the protein surface, which connects the pore and the cytoplasm, as described earlier (Supplementary Figure 11). The hydrophilic 'head' of the lipid is well-resolved at high resolution and interacts directly with water molecules of the intracellular cavity (Supplementary Figure 11). We suspect that the pore is accidentally blocked by the lipid molecule of the host crystallization matrix. In order to address the potential structural importance of the lipid molecule and the stability of the OLPVR1 in its absence, we performed molecular dynamics simulations of OLPVR1 without the lipid fragment. During the simulation, protein did not show any significant side chain displacement in the nearest vicinity of the lipid-binding region (Supplementary Figure 11d). In addition, we designed a variant of the OLPVR1 with nine mutations (I169L, Y172G, F173A, V177L, F179A, V202F, I206F, Y207V, I211F) that we denote as O1O2 protein, which intracellular parts of TM6 and TM7 helices are supposed to mimic those of OLPVRII from VR2 group[1] (Supplementary Figure 11c). We expressed, purified, and crystallized O1O2 similarly to the wild type protein. O1O2 crystals diffracted up to 1.9 Å and contained two protein molecules in the asymmetric unit, organized into the same dimer as demonstrated for the wild type OLPVR1 (Supplementary Figure 9). Interestingly, although the pore in the intracellular part of the O1O2 is similar to that of OLPVR1, we observed no lipid fragments in the lipid-binding region (Supplementary Figure 11b). Thus, lipid molecule most likely does not play a role in structural stabilization of the protein, but rather employ loose configuration between TM6 and TM7 helices and presumably is a crystallization artifact.

Comparison of the structures of group 1 viral rhodopsins

Recently, another viral rhodopsin of the VR1 group ($VirR_{DTS}$) was reported[2]. It shares high sequence similarity with OLPVR1 and VirChR1 in terms of functionally important residues. Surprisingly, it was reported to be a proton pump. To verify its function, we expressed $VirR_{DTS}$ in HEK293 cells and conducted voltage-clamp measurements (Supplementary Figure 13e). Predictably, the data show photocurrents, which reverse their direction at approximately 0 mV,

which is characteristic of rhodopsin ion channels, but not of the ion pumps. Based on these data, we conclude that $VirR_{DTS}$ is also a light-gated channel. To underline similarities and discrepancies between two available structures of viral rhodopsins from group 1, we performed a detailed comparison of OLPVR1 (present work) and $VirR_{DTS}$ (PDB ID: 6J0O[2]). The structures present nearly identical topology of the helices and superimpose with RMSD of 0.44 Å (Supplementary Figure 13). The ICL2 is found in both OLPVR1 and $VirR_{DTS}$ and is organized similarly in the proteins. Similar to other viral rhodopsins from group 1, $VirR_{DTS}$ conserves all residues that are predicted to be responsible for the ion-channeling activity of VirChR1. In particular, we observed nearly identical orientations of side chains of S11, E44, E51, N197, and N205 residues in both structures, suggesting a similar configuration of those residues among all VirChR1s. The hydrogen bonding network, connecting the ICS and CCS, is found in both OLPVR1 and $VirR_{DTS}$, however, with minor differences. For instance, there are more water molecules in $VirR_{DTS}$ in the region between E47 and E54 (E44 and E51 in OLPVR1, respectively). This is likely due to the substitution of serine (S208 in OLPVR1) to smaller amino acid residue glycine (G215 in $VirR_{DTS}$). Therefore, the hydrogen bonding network between ICS and CCS is mediated by waters to a greater extent in $VirR_{DTS}$ than in OLPVR1.

Interestingly, $VirR_{DTS}$ contains a lipid molecular fragment between helices TM6 and TM7 similar to that found in OLPVR1 structures, supporting the hypothesis on the conservation of the pore structure between TM6 and TM7 in viral rhodopsins of group 1. Taken together, this additionally supports the conclusion that the lipid molecule is an artifact of crystallization.

Supplementary Figures

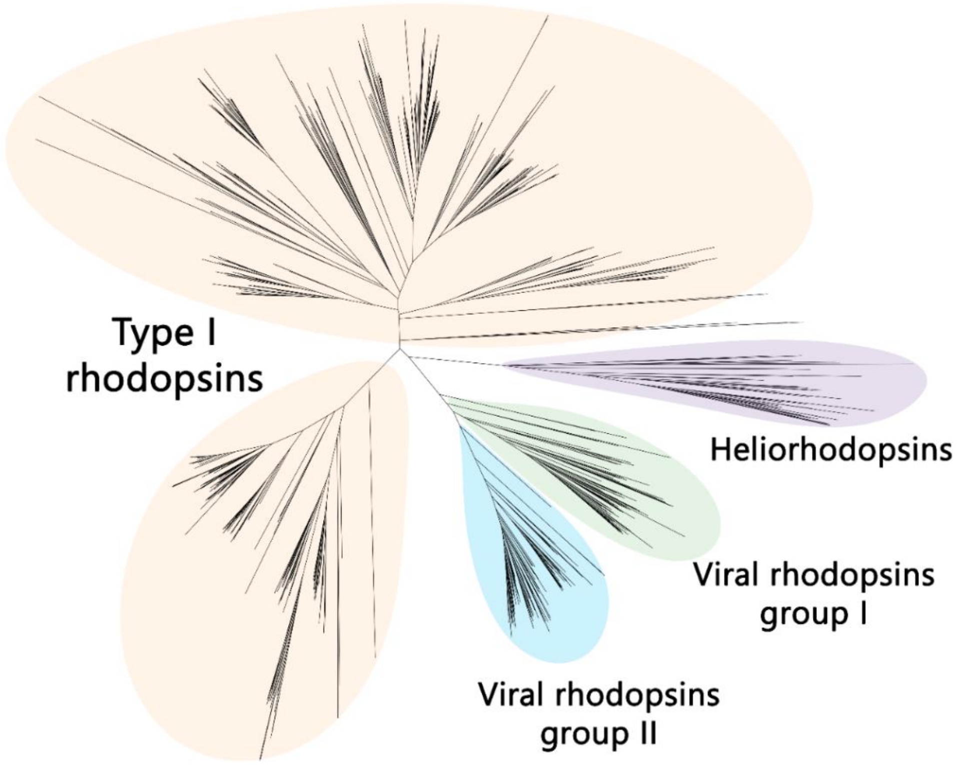

Supplementary Figure 1. Unrooted phylogenetic tree of known microbial rhodopsins. Viral rhodopsins (groups I and II), heliorhodopsins[3,4], and other microbial rhodopsins (type-I rhodopsins) groups are indicated with color. The analyzed base of microbial rhodopsins consists of proteins retrieved from TARA project and proteins predicted with InterPro[5] software. The phylogenetic tree was created using UGENE[6] and iTOL[7] software.

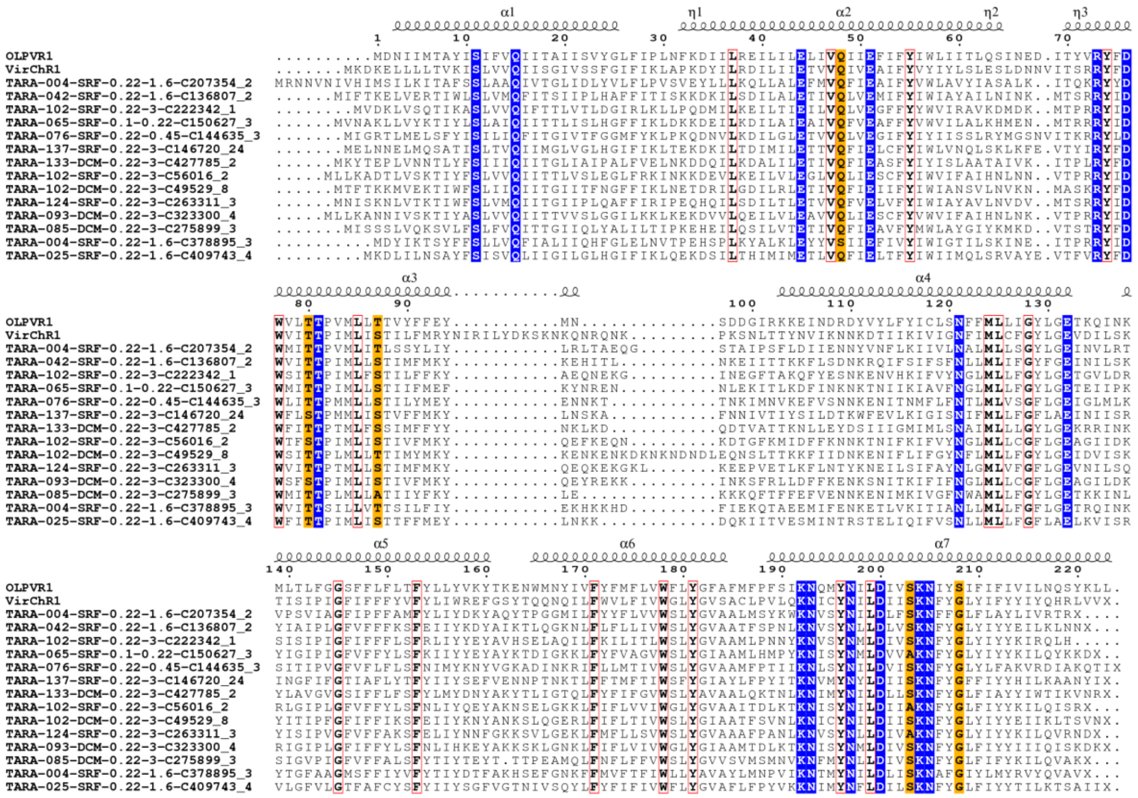

Supplementary Figure 2. Sequence alignment of representative viral rhodopsins. The sequence alignment was created using UGENE software[6] and ESPript3 online server. Secondary structure elements for OLPVR1 are shown as coils. Fully conservative residues are indicated with blue color and red frames for charged and non-charged amino acids, respectively. Highly conservative residues forming ion channeling constrictions are additionally colored orange.

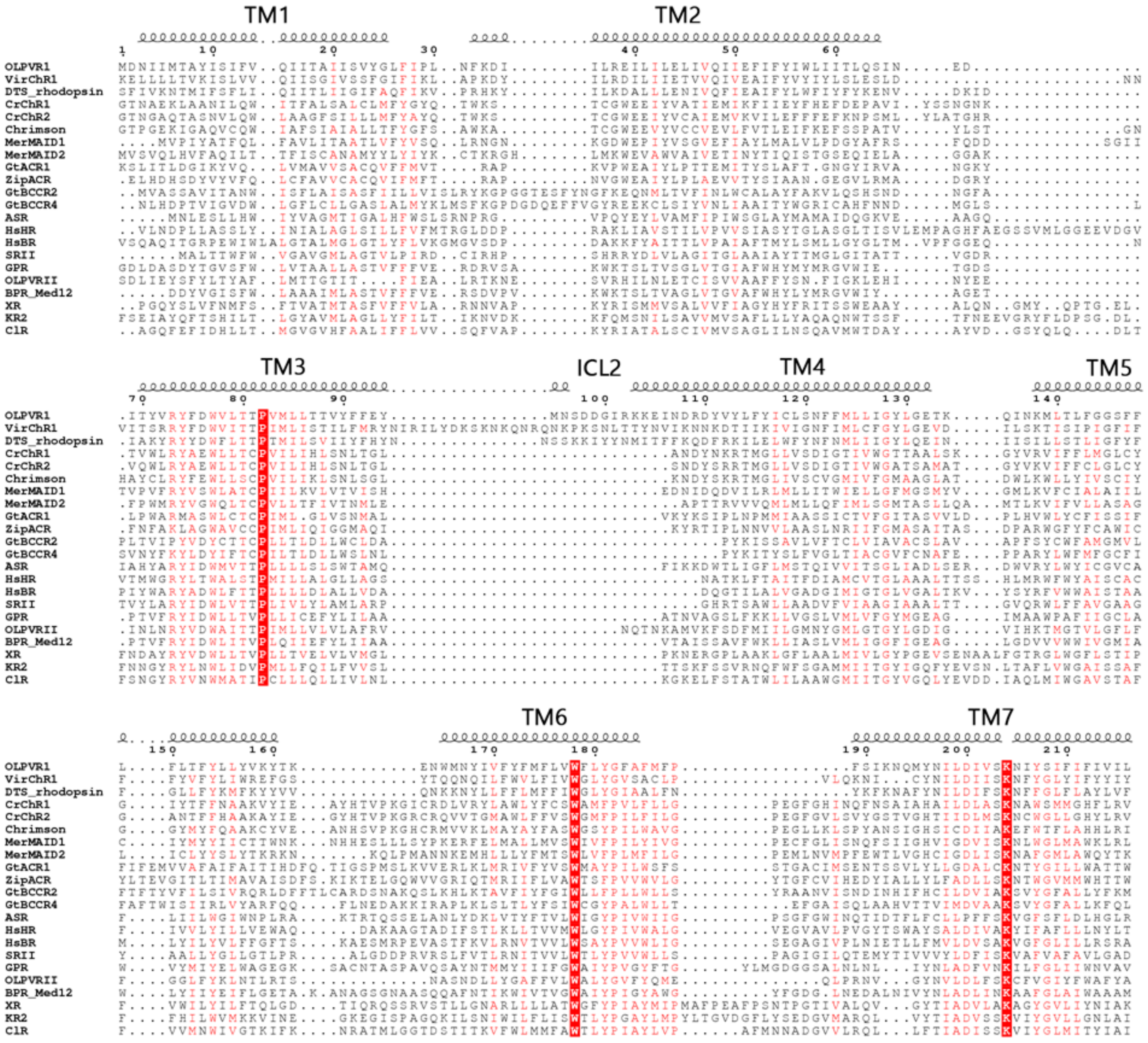

Supplementary Figure 3. Sequence alignment of microbial rhodopsin genes. The sequences with their Genbank IDs in parenthesis are OLPVR1 (ADX06642.1, present study), VirChR1 (TARA-146-SRF-0.22-3-C376786_1, present study), DTS rhodopsin (AGM15640.1), Chrimson (AHH02126.1), *Cr*ChR2 (ABO64386.1), MerMAID1[8], MerMAID284[8], *Gt*ACR1 (AKN63094.1), ZipACR (APZ76709.1), *Gt*BCCR2 (ANC73518.1), *Gt*BCCR4 (ARQ20888.1), *Hs*BR (WP_010903069.1), *Hs*HR (P15647.1), ClR (PDB ID: 5G28), KR2 (BAN14808.1), GPR (AAK30191.1), BPR_*Med12* (PDB ID: 4JQ6). Highly conservative residues are colored red, and fully conservative residues are indicated with red frames.

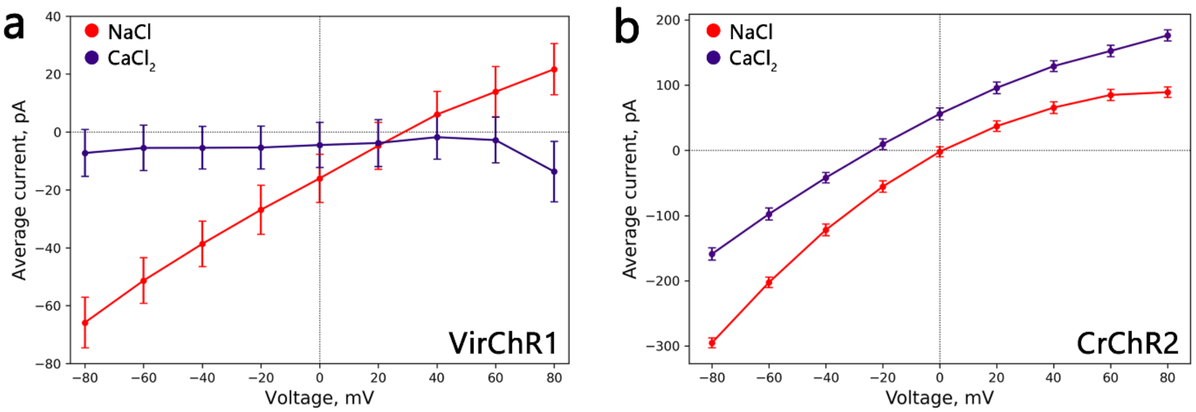

Supplementary Figure 4. Comparison of calcium sensitivity of VirChR1 and *Cr*ChR2 (a) Current-voltage dependences for one representative SH-SY5Y cell in 110 mM NaCl (red) and 80 mM $CaCl_2$ (indigo) solutions. Error bars were determined as a standard deviation of current value under illumination in the cell measured. No additional averaging was done, since different cells have different protein expression levels. For both proteins Sample size n = 1 for data displayed. Current-traces had similar shape in n = 3 - 10 more cells. (b) Representative example of current-voltage dependences of a *Cr*ChR2-expressing SH-SY5Y cell in 80 mM $CaCl_2$ solution. We did not observe calcium blocking effect of *Cr*ChR2 in any cell.

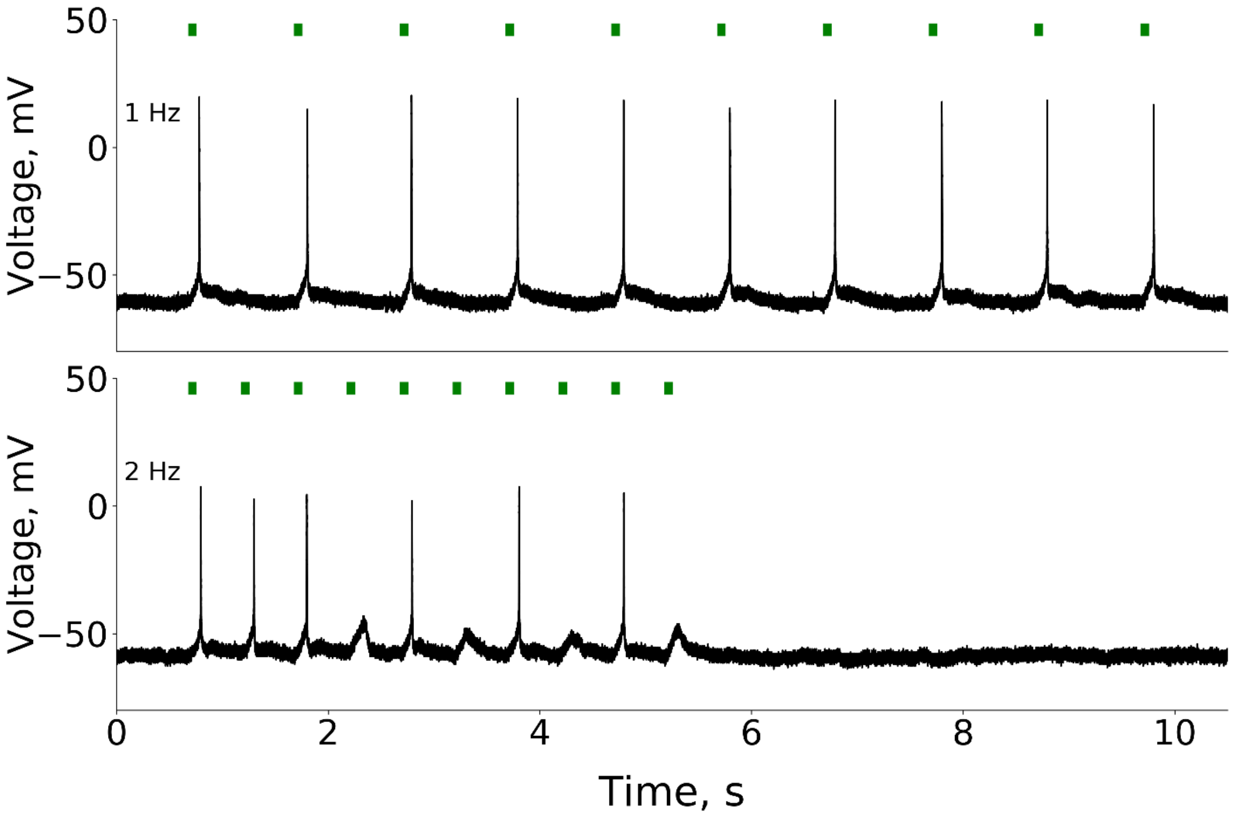

Supplementary Figure 5. Voltage-traces of one representative neuron spiking in response to 80 ms light pulse series of different frequencies. Missed spikes occur in higher frequency.

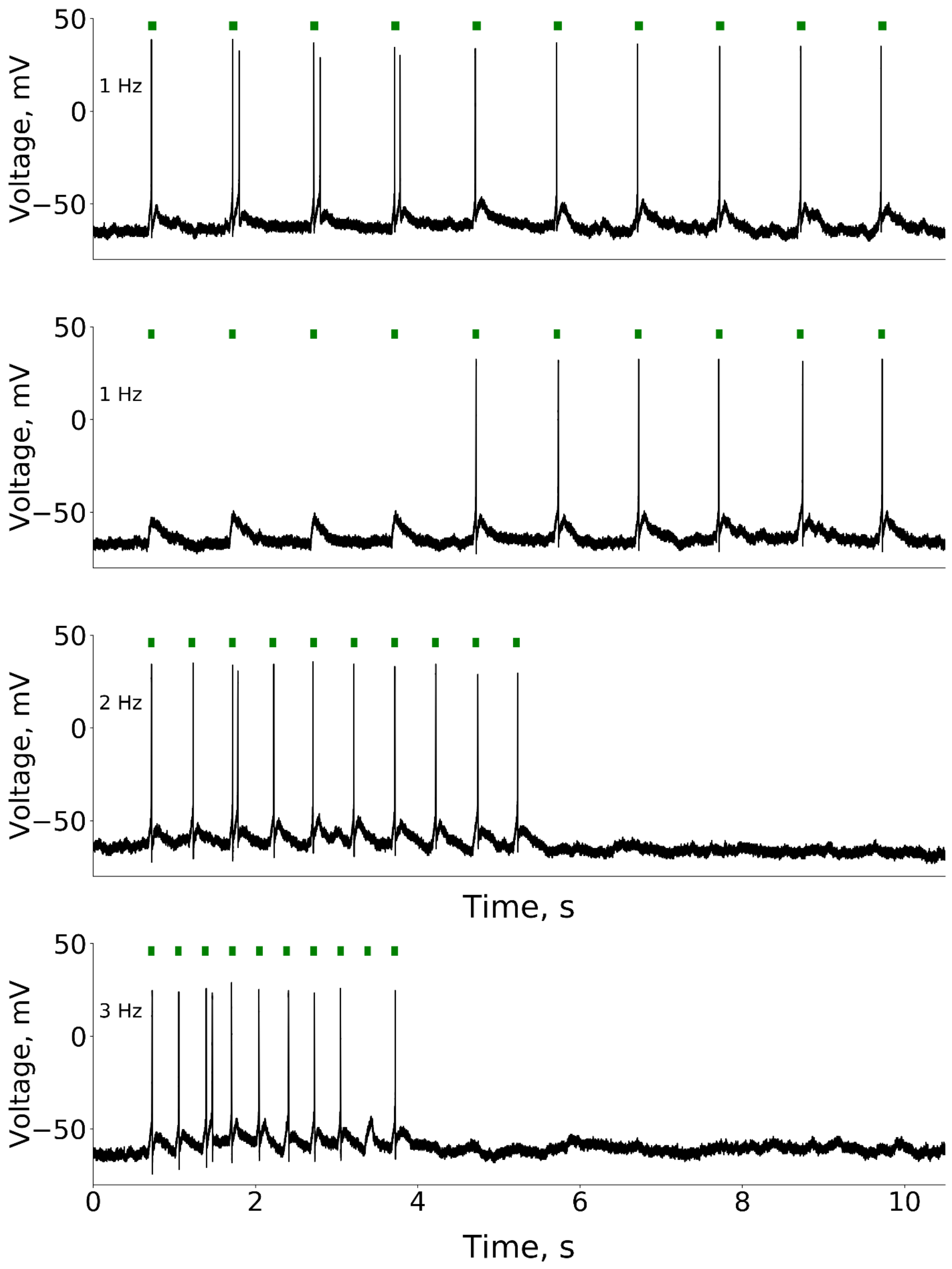

Supplementary Figure 6. Representative examples of VirChR1 neuron spiking. Voltage-traces of one representative neuron spiking in response to light pulse series (top – 100 ms light pulses, others – 80 ms light pulses). Double-spiking occurs in some cases.

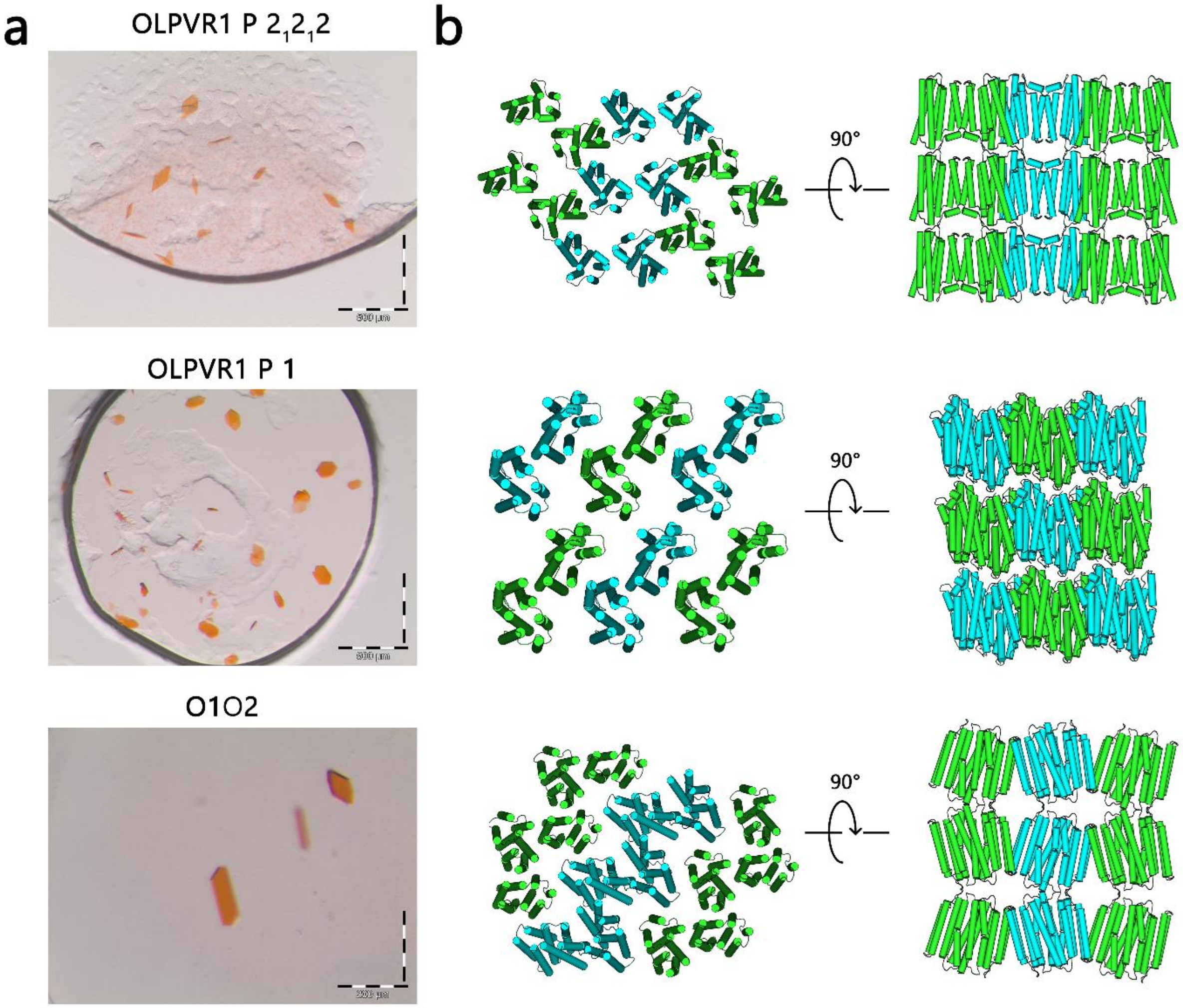

Supplementary Figure 7. Crystal packing of OLPVR1 protein. (a) Magnified images of the OLPVR1 and O1O2 crystals used for structure determination. Crystals were observed in at least n = 2 independent experiments, and under n = 2-7 slightly different precipitation buffer conditions for each experiment. (b) Lattice packing of the crystals, viewed in two principal orientations. The asymmetric unit protein content is colored with green and cyan for each structure.

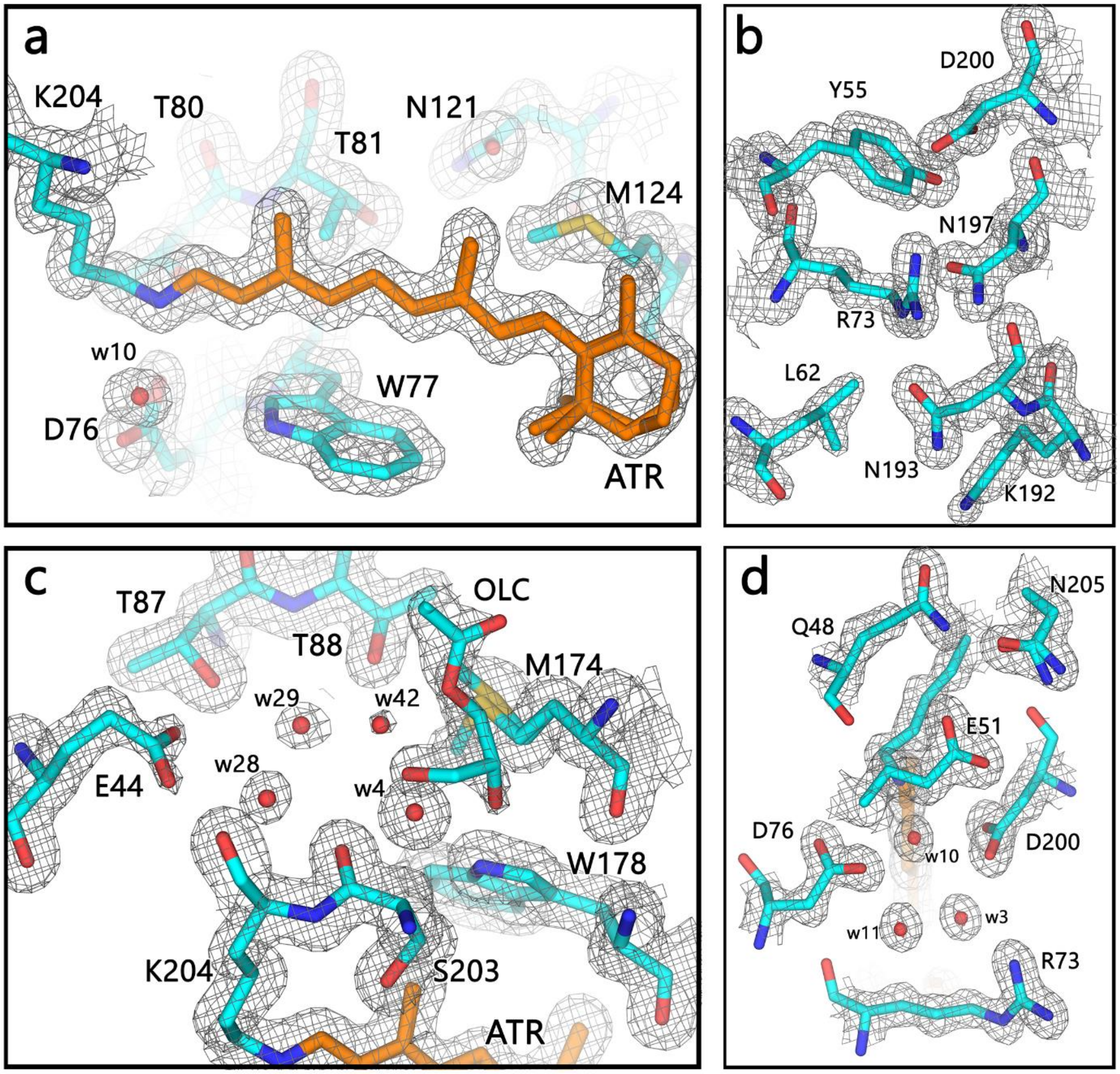

Supplementary Figure 8. Examples of the electron density maps of the OLPVR1 structure. $2F_o - F_c$ maps (gray mesh, contoured at the level of 1.5σ) for the (a) retinal pocket, (b) intracellular gate, (c) extracellular gate, and (d) central gate. The water molecules and retinal cofactor are clearly resolved in the structure. 1.4 Å resolution dataset was collected with a single crystal.

a

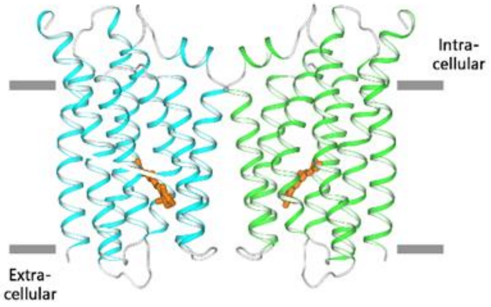

b

c

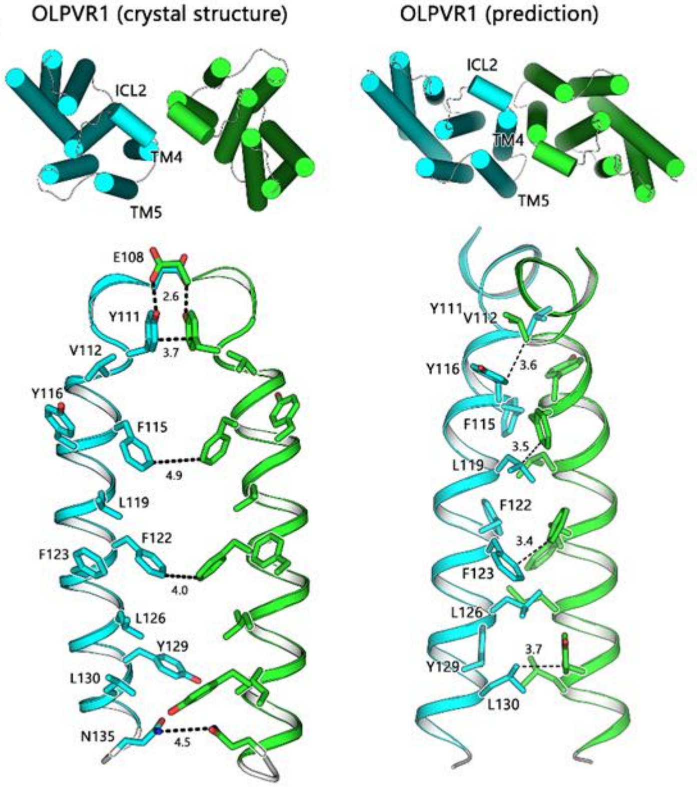

d

	Interaction surface area (Å²)
OLPVR1 (crystal packing)	429
OLPVR1 (prediction)	1861
HsBR trimer	1417
CrChR2 dimer	3441

Supplementary Figure 9. Putative dimer organization of OLPVR1. (a) Side view and the view from the extracellular side of the OLPVR1 putative dimer observed for $P2_12_12$ structure. (b) OLPVR1 crystal structure (left) and OLPVR1 dimer structure predicted by GalaxyHomomer server[9] (right). (c) Corresponding dimer interface of dimer structures formed by ICL2, TM4 and TM5 helices. Distances between residues involved in dimer formation are drawn with dashed lines. Residues involved into dimer formation are named in one of the protomers. (d) Interaction surface areas between protomers in different microbial rhodopsins structures calculated with solvent accessible surface area calculation software for biological molecules.

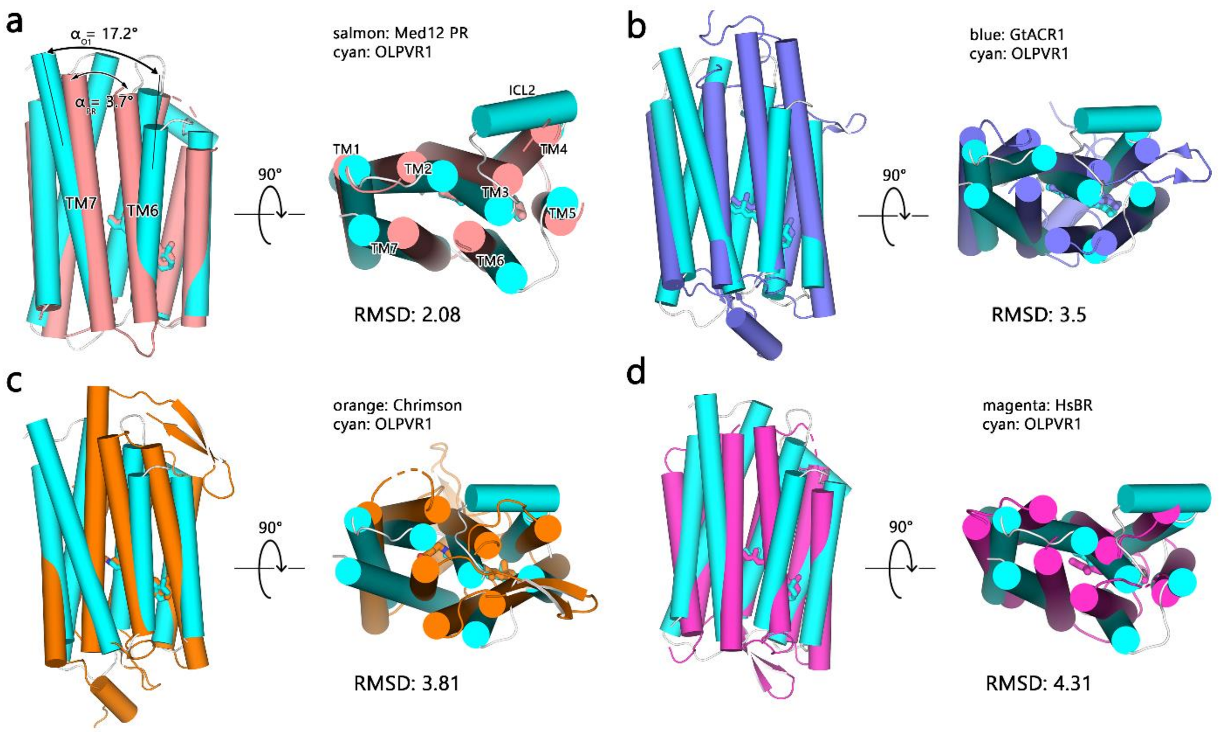

Supplementary Figure 10. Structural alignment of OLPVR1 with other rhodopsins. Side views and views from extracellular side of pair structure alignments of OLPVR1 with (a) *Med12*BPR (PDB ID: 4JQ6[10]), (b) *Gt*ACR1 (PDB: 6CSM[11]), (c) Chrimson (PDB: 5ZIH[12]) and (d) *Hs*BR (PDB: 1C3W[13]). Root mean square deviations (RMSD) of the alignments are additionally indicated.

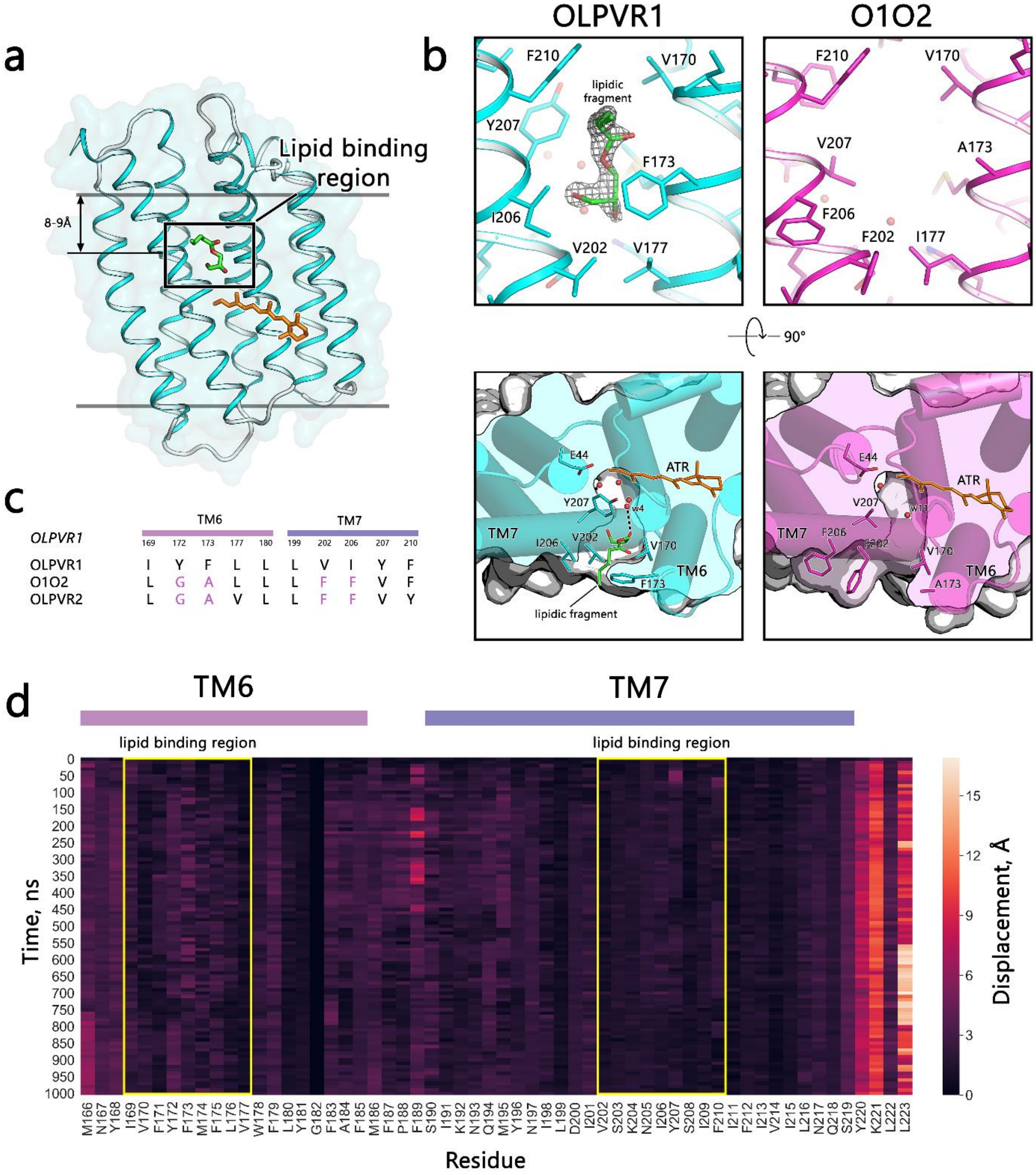

Supplementary Figure 11. Unusual OLPVR1-lipid interaction. (a) Lipid binding region (LBR) in the OLPVR1 structure between TM6 and TM7 helices. The lipidic fragment is drawn as green sticks. (b) The magnified view of the protein-lipid interaction region in OLPVR1 (left) and O1O2 (right). $2F_o - F_c$ map of the lipidic fragment between TM6 and TM7 helices is contoured at 1.5σ and shown as gray mesh. No lipidic fragment in the LBR in O1O2 structure was observed. (c) Sequence alignment of the TM6 and TM7 helices of OLPVR1, OLPVRII[14], and O1O2 mutant. Major mutations implemented are colored purple. (d) Heat plot of side chain displacement (root mean square deviation, RMSD) of residues from TM6 and TM7 at 1 μs scale after lipid fragment (97N 302 ligand molecule) removal.

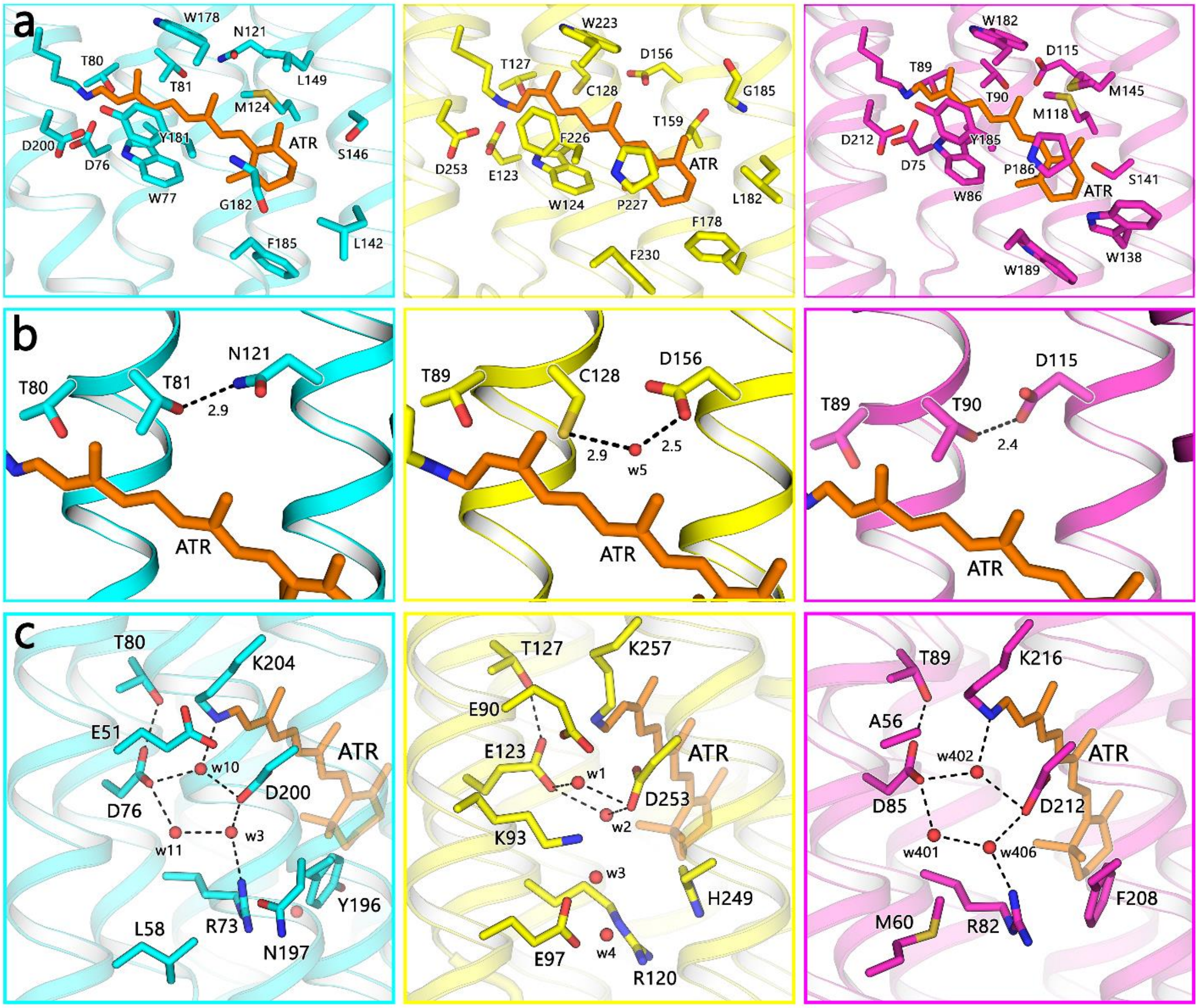

Supplementary Figure 12. Retinal pocket and DC pair region architecture. (a) Retinal pockets, (b) DC pair regions, and (c) Schiff base regions of OLPVR1 (left), *Cr*ChR2 (middle), and *Hs*BR (right) are shown in cyan, yellow, and purple colors respectively. All-*trans* retinal cofactor is depicted orange. Essential residues are numbered and depicted as sticks. Hydrogen bonds are shown with dashed lines. Distances between DC-pair partnering residues are indicated in angstroms.

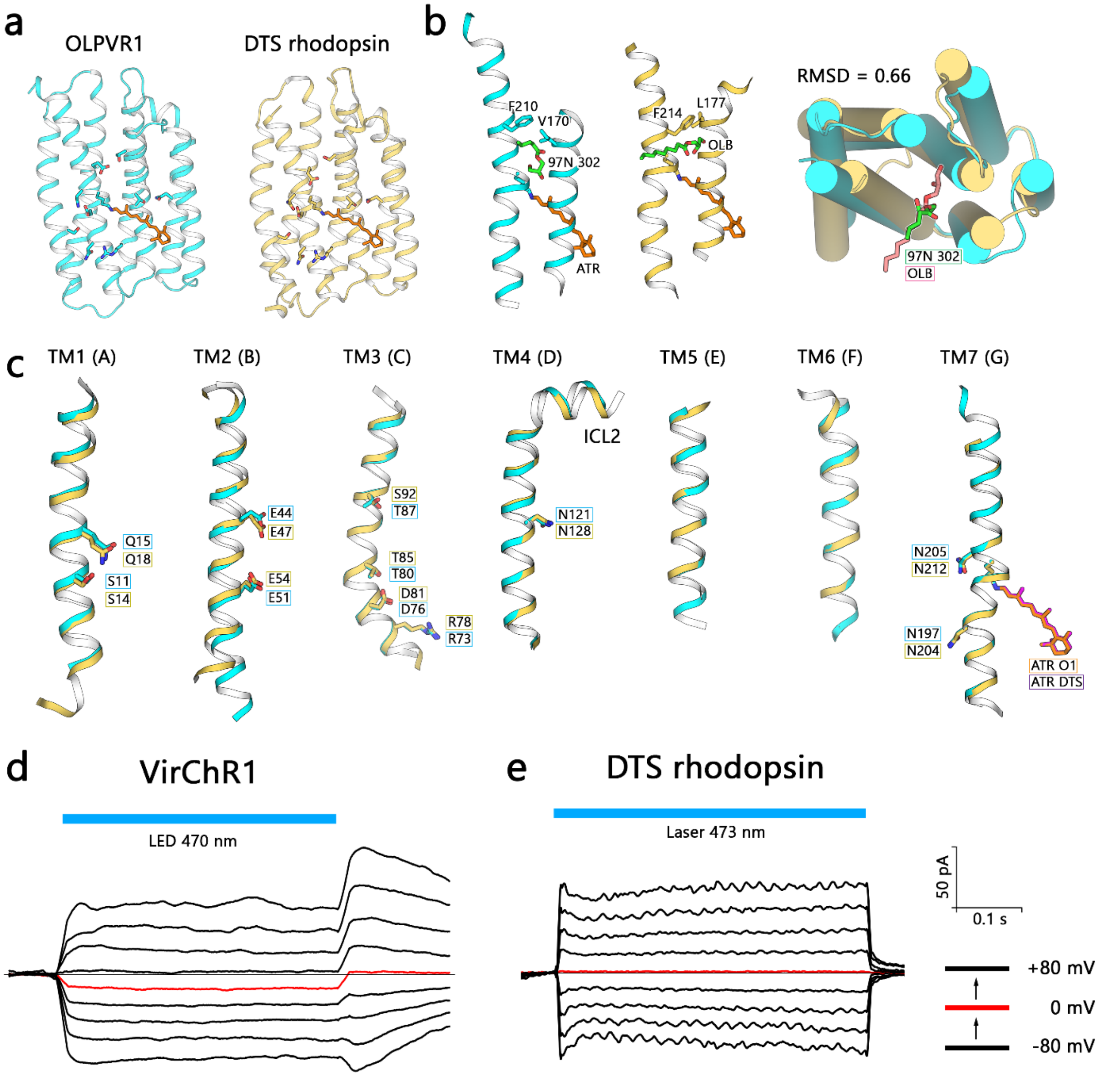

Supplementary Figure 13. Comparison of OLPVR1 and VirR$_{DTS}$. (a) Crystal structures of OLPVR1 and VirR$_{DTS}$ (PDB: 6JO0[2]). (b) An extended overview of the lipid molecule fragment orientation between TM6 and TM7 helices in viral rhodopsins structures viewed parallel to membrane (left) and from the intracellular side (right). (c) Individual TM helices are shown after the superimposition of the OLPVR1 and VirR$_{DTS}$. Key residues are additionally shown as sticks. (d) Voltage-clamp records from one representative cell, expressing (d) VirChR1 (left) and VirR$_{DTS}$ (DTS rhodopsin) with 10 mM HEPES pH 7.4, 110 mM NaCl, 2 mM $MgCl_2$. The illumination period is indicated in light blue.

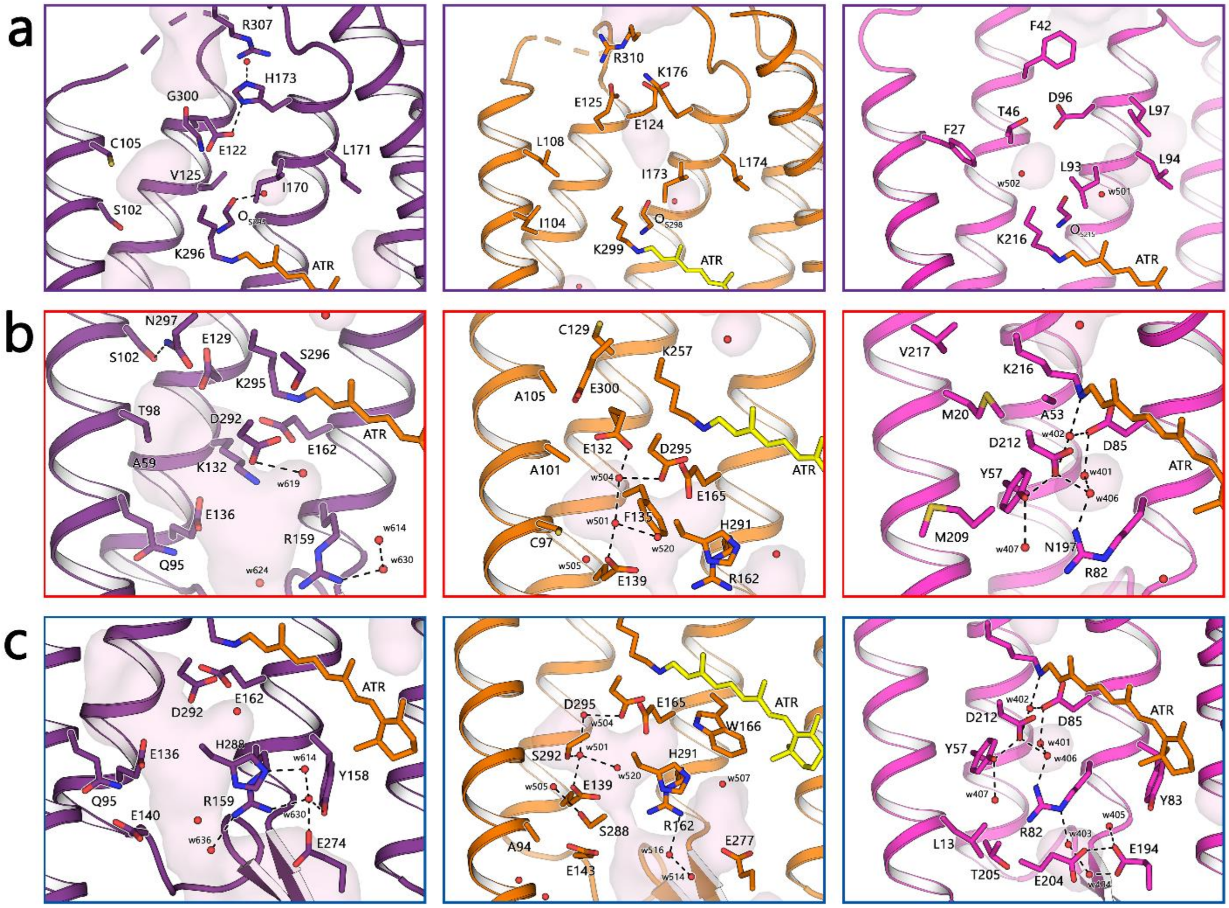

Supplementary Figure 14. Organization of ion pathway constriction sites in other microbial rhodopsins. Magnified view of the (a) intracellular constriction site (gate), (b) central constriction site (gate) and (c) extracellular constriction site (gate) regions in C1C2 (left, PDB code: 3UG9[15]), Chrimson (middle, PDB code: 5ZIH[12]) and *Hs*BR (right, PDB code: 1C3W[13]) structures, colored indigo, orange and magenta respectively. Water accessible cavities were calculated using HOLLOW[16] and are presented as pink surfaces.

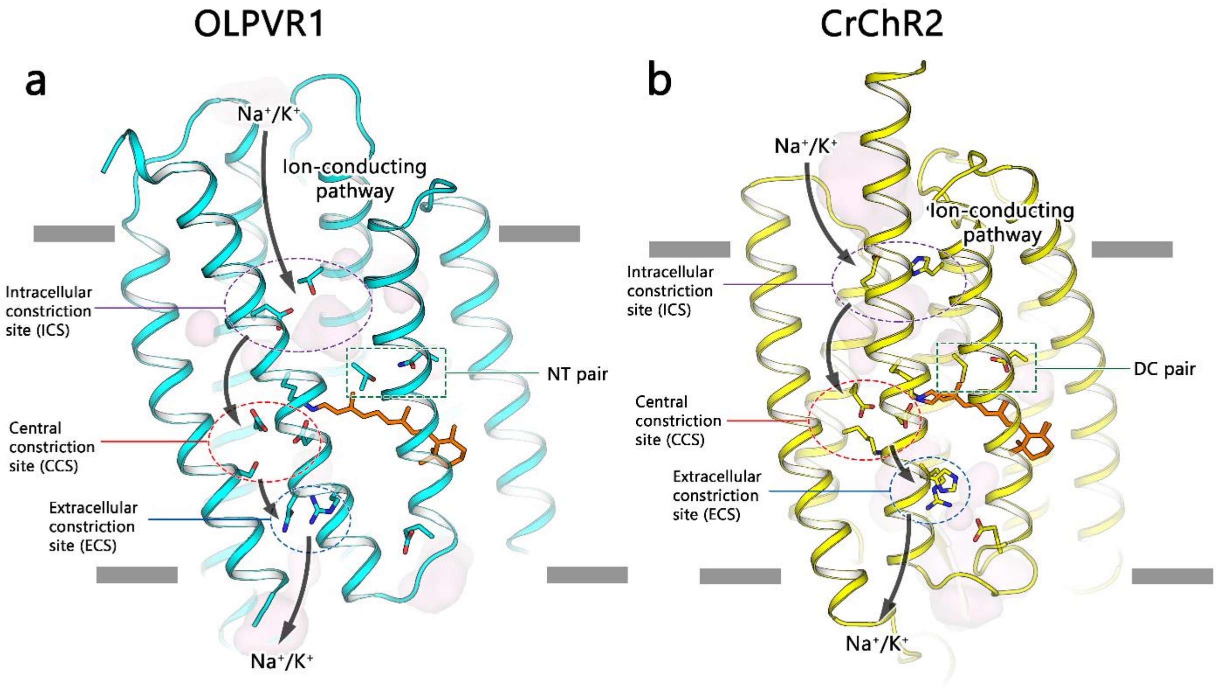

Supplementary Figure 15. Proposed ion-conducting pathway of OLPVR1. Three consecutive constriction sites and proposed cation pathway of (a) OLPVR1 and (b) *Cr*ChR2 proteins. The proposed ion pathway is shown only in one direction for clarity. Important residues are depicted as sticks. All-*trans* retinal is painted with orange color. NT and DC pairs are additionally indicated.

Supplementary Table 1. Crystallographic data collection and refinement statistics.

**Data collection**	OLPVR1_P1	OLPVR1_P21212	O1O2
Space group	P 1	P 2_1 2_1 2	I 1 2 1
Cell dimensions			
a, *b*, *c* (Å)	40.18, 56.97, 62.34	46.53, 115.80, 53.53	44.40, 103.40, 111.38
α, β, γ (º)	113.90, 90.01, 91.49	90, 90, 90	90, 90.32, 90
Wavelength (Å)	0.976	0.976	0.978
Resolution (Å)	40.16-1.6 (1.63-1.60)	48.59-1.4 (1.42-1.40)	41.33-1.96 (2.00-1.96)
R_{merge} (%)	5.8 (116.1)	7.2 (181.0)	5.1 (119.1)
$I/\sigma I$	6.1 (0.7)	12.3 (1.0)	12.2 (0.9)
$CC_{1/2}$ (%)	99.8 (53.3)	99.8 (45.3)	99.9 (50.8)
Completeness (%)	95.2 (94.2)	100.0 (99.9)	99.5 (95.4)
Unique reflections	63,486 (3098)	57,845 (2820)	36,098 (2448)
Multiplicity	3.5 (3.6)	6.3 (6.3)	3.5 (3.4)
**Refinement**			
Resolution (Å)	20-1.60	20-1.40	20-1.96
No. reflections	60,315	54,864	34,293
$R_{work/}$ R_{free} (%)	18.8/21.3	14.2/18.6	20.2/24.6
No. atoms			
Protein	3915	2012	3776
Retinal	40	40	40
Water	98	107	52
Lipids	289	220	32
B-factors (Å²)			
Protein	31	24	45
Retinal	23	17	35
Water	36	37	42
Lipids	54	46	58
R.m.s. deviations			
Bond lengths (Å)	0.0030	0.0041	0.0021
Bond angles (º)	1.0662	0.8917	1.0525

Supplementary Table 2. List of plasmid used in this study.

pBKT plasmid<br>(gene position<br>described as XXX)	agatcttgatcccctgcgccatcagatccttggcggcaagaaagccatccagtttactttgcagggcttcccaaccttaccagagggcgccccagctggcaattccgggccacgatgcgtccggcgtagag<br>gatcgagatctcgatcccgcgaaattaatacgactcactataggggaattgtgagcggataacaattcccctctagaaataattttgtttaactttaagaaggagatatacatXXXXXXXXXXXXXX<br>XXXXgagagtggtctggtgccgcgcggcagcggtcaccaccatcaccaccatcaccactaatgaaagcttggctgctaacaaagcccgaaaggaagctgagttggctgctgccaccgctgagcaata<br>actagcataaccccttggggcctctaaacgggtcttgaggggttttttgctgaaaggaggaactatatccggcgcgtgctagaggcatcaaataaaacgaaaggctcagtcgaaagactgggcctttcgtttta<br>tctgttgtttgtcggtgaacgctctcctgagtaggacaaatccgccgccctagacctagggcgttcggctgcggcgagcggtatcagctcactcaaaggcggtaatacggttatccacagaatcaggggata<br>acgcaggaaagaacatgtgagcaaaaggccagcaaaaggccaggaaccgtaaaaaggccgcgttgctggcgtttttccataggctccgcccccctgacgagcatcacaaaaatcgacgctcaagtcaga<br>ggtggcgaaacccgacaggactataaagataccaggcgtttccccctggaagctccctcgtgcgctctcctgttccgaccctgccgcttaccggatacctgtccgcctttctcccttcgggaagcgtggcgct<br>ttctcatagctcacgctgtaggtatctcagttcggtgtaggtcgttcgctccaagctgggctgtgtgcacgaaccccccgttcagcccgaccgctgcgccttatccggtaactatcgtcttgagtccaacccggt<br>aagacacgacttatcgccactggcagcagccactggtaacaggattagcagagcgaggtatgtaggcggtgctacagagttcttgaagtggtggcctaactacggctacactagaaggacagtatttggtat<br>ctgcgctctgctgaagccagttaccttcggaaaaagagttggtagctcttgatccggcaaacaaaccaccgctggtagcggtggtttttttgtttgcaagcagcagattacgcgcagaaaaaaaggatctcaag<br>aagatcctttgatcttttctacggggtctgacgctcagtggaacgaaaactcacgttaagggattttggtcatgactagtgcttggattctcaccaataaaaaacgcccggcggcaaccgagcgttctgaacaaa<br>tccagatggagttctgaggtcattactggatctatcaacaggagtccaagcgagctctcgaaccccagagtcccgctcagaagaactcgtcaagaaggcgatagaaggcgatgcgctgcgaatcgggagc<br>ggcgataccgtaaagcacgaggaagcggtcagcccattcgccgccaagctcttcagcaatatcacgggtagccaacgctatgtcctgatagcggtccgccacacccagccggccacagtcgatgaatcca<br>gaaaagcggccattttccaccatgatattcggcaagcaggcatcgccatgggtcacgacgagatcctcgccgtcgggcatgcgcgccttgagcctggcgaacagttcggctggcgcgagcccctgatgct<br>cttcgtccagatcatcctgatcgacaagaccggcttccatccgagtacgtgctcgctcgatgcgatgtttcgcttggtggtcgaatgggcaggtagccggatcaagcgtatgcagccgccgcattgcatcagc<br>catgatggatactttctcggcaggagcaaggtgagatgacaggagatcctgccccggcacttcgcccaatagcagccagtcccttcccgcttcagtgacaacgtcgagcacagctgcgcaaggaacgccc<br>gtcgtggccagccacgatagccgcgctgcctcgtcctgcagttcattcagggcaccggacaggtcggtcttgacaaaaagaaccgggcgcccctgcgctgacagccggaacacggcggcatcagagca<br>gccgattgtctgttgtgcccagtcatagccgaatagcctctccacccaagcggccggagaacctgcgtgcaatccatcttgttcaatcatgcgaaacgatcctcatcctgtctcttgatc
pCDNA3.1 plasmid<br><br>(gene position<br>described as XXX)	atctcccgatcccctatggtgcactctcagtacaatctgctctgatgccgcatagttaagccagtatctgctccctgcttgtgtgttggaggtcgctgagtagtgcgcgagcaaaatttaagctacaacaaggcaa<br>ggcttgaccgacaattgcatgaagaatctgcttagggttaggcgttttgcgctgcttcgcgatgtacgggccagatatacgcgttgacattgattattgactagttattaatagtaatcaattacggggtcattagtt<br>catagcccatatatggagttccgcgttacataacttacggtaaatggcccgcctggctgaccgcccaacgacccccgcccattgacgtcaataatgacgtatgttcccatagtaacgccaatagggactttcca<br>ttgacgtcaatgggtggagtatttacggtaaactgcccacttggcagtacatcaagtgtatcatatgccaagtacgccccctattgacgtcaatgacggtaaatggcccgcctggcattatgcccagtacatgac<br>cttatgggactttcctacttggcagtacatctacgtattagtcatcgctattaccatggtgatgcggttttggcagtacatcaatgggcgtggatagcggtttgactcacggggatttccaagtctccaccccattga<br>cgtcaatgggagtttgttttggcaccaaaatcaacgggactttccaaaatgtcgtaacaactccgccccattgacgcaaatgggcggtaggcgtgtacggtgggaggtctatataagcagagctctctggcta<br>actagagaacccactgcttactggcttatcgaaattaatacgactcactatagggagacccaagctggctagcgtttaaacgggccctctagactcgagtggccgccactgtgctggatatctgcagaattcca<br>ccacactggactagtggatccgccaccXXXXXXXXXXXXXXXXXXcttaagtttaaaccgctgatcagcctcgactgtgccttctagttgccagccatctgttgtttgcccctcccccgtgcc<br>ttccttgaccctggaaggtgccactcccactgtcctttcctaataaaatgaggaaattgcatcgcattgtctgagtaggtgtcattctattctggggggtggggtggggcaggacagcaagggggaggattggg<br>aagacaatagcaggcatgctggggatgcggtgggctctatggcttctgaggcggaaagaaccagctggggctctagggggtatccccacgcgccctgtagcggcgcattaagcgcggcgggtgtggtg<br>gttacgcgcagcgtgaccgctacacttgccagcgccctagcgcccgctcctttcgctttcttcccttcctttctcgccacgttcgccggctttccccgtcaagctctaaatcgggggctccctttagggttccgatt<br>tagtgctttacggcacctcgaccccaaaaaacttgattagggtgatggttcacgtagtgggccatcgccctgatagacggtttttcgccctttgacgttggagtccacgttctttaatagtggactcttgttccaaac<br>tggaacaacactcaaccctatctcggtctattcttttgatttataagggattttgccgatttcggcctattggttaaaaaatgagctgatttaacaaaaatttaacgcgaattaattctgtggaatgtgtgtcagttaggg<br>tgtggaaagtccccaggctccccagcaggcagaagtatgcaaagcatgcatctcaattagtcagcaaccaggtgtggaaagtccccaggctccccagcaggcagaagtatgcaaagcatgcatctcaatta<br>gtcagcaaccatagtcccgcccctaactccgcccatcccgcccctaactccgcccagttccgcccattctccgccccatggctgactaattttttttatttatgcagaggccgaggccgcctctgcctctgagcta<br>ttccagaagtagtgaggaggcttttttggaggcctaggcttttgcaaaaagctcccgggagcttgtatatccattttcggatctgatcaagagacaggatgaggatcgtttcgcatgattgaacaagatggattgc<br>acgcaggttctccggccgcttgggtggagaggctattcggctatgactgggcacaacagacaatcggctgctctgatgccgccgtgttccggctgtcagcgcaggggcgcccggttctttttgtcaagaccg<br>acctgtccggtgccctgaatgaactgcaggacgaggcagcgcggctatcgtggctggccacgacgggcgttccttgcgcagctgtgctcgacgttgtcactgaagcgggaagggactggctgctattggg<br>cgaagtgccggggcaggatctcctgtcatctcaccttgctcctgccgagaaagtatccatcatggctgatgcaatgcggcggctgcatacgcttgatccggctacctgcccattcgaccaccaagcgaaacat<br>cgcatcgagcgagcacgtactcggatggaagccggtcttgtcgatcaggatgatctggacgaagagcatcaggggctcgcgccagccgaactgttcgccaggctcaaggcgcgcatgcccgacggcga<br>ggatctcgtcgtgacccatggcgatgcctgcttgccgaatatcatggtggaaaatggccgcttttctggattcatcgactgtggccggctgggtgtggcggaccgctatcaggacatagcgttggctacccgt<br>gatattgctgaagagcttggcggcgaatgggctgaccgcttcctcgtgctttacggtatcgccgctcccgattcgcagcgcatcgccttctatcgccttcttgacgagttcttctgagcgggactctggggttcg<br>aaatgaccgaccaagcgacgcccaacctgccatcacgagatttcgattccaccgccgccttctatgaaaggttgggcttcggaatcgttttccgggacgccggctggatgatcctccagcgcggggatctca<br>tgctggagttcttcgcccaccccaacttgtttattgcagcttataatggttacaaataaagcaatagcatcacaaatttcacaaataaagcatttttttcactgcattctagttgtggtttgtccaaactcatcaatgtatc<br>ttatcatgtctgtataccgtcgacctctagctagagcttggcgtaatcatggtcatagctgtttcctgtgtgaaattgttatccgctcacaattccacacaacatacgagccggaagcataaagtgtaaagcctggg<br>gtgcctaatgagtgagctaactcacattaattgcgttgcgctcactgcccgctttccagtcgggaaacctgtcgtgccagctgcattaatgaatcggccaacgcgcggggagaggcggtttgcgtattgggcg<br>ctcttccgcttcctcgctcactgactcgctgcgctcggtcgttcggctgcggcgagcggtatcagctcactcaaaggcggtaatacggttatccacagaatcaggggataacgcaggaaagaacatgtgagc<br>aaaaggccagcaaaaggccaggaaccgtaaaaaggccgcgttgctggcgtttttccataggctccgcccccctgacgagcatcacaaaaatcgacgctcaagtcagaggtggcgaaacccgacaggact<br>ataaagataccaggcgtttccccctggaagctccctcgtgcgctctcctgttccgaccctgccgcttaccggatacctgtccgcctttctcccttcgggaagcgtggcgctttctcatagctcacgctgtaggtat<br>ctcagttcggtgtaggtcgttcgctccaagctgggctgtgtgcacgaaccccccgttcagcccgaccgctgcgccttatccggtaactatcgtcttgagtccaacccggtaagacacgacttatcgccactgg<br>cagcagccactggtaacaggattagcagagcgaggtatgtaggcggtgctacagagttcttgaagtggtggcctaactacggctacactagaagaacagtatttggtatctgcgctctgctgaagccagttac<br>cttcggaaaaagagttggtagctcttgatccggcaaacaaaccaccgctggtagcggtttttttgtttgcaagcagcagattacgcgcagaaaaaaaggatctcaagaagatcctttgatcttttctacggggtct<br>gacgctcagtggaacgaaaactcacgttaagggattttggtcatgagattatcaaaaaggatcttcacctagatccttttaaattaaaaatgaagttttaaatcaatctaaagtatatatgagtaaacttggtctgaca<br>gttaccaatgcttaatcagtgaggcacctatctcagcgatctgtctatttcgttcatccatagttgcctgactccccgtcgtgtagataactacgatacgggagggcttaccatctggccccagtgctgcaatgata<br>ccgcgagacccacgctcaccggctccagatttatcagcaataaaccagccagccggaagggccgagcgcagaagtggtcctgcaactttatccgcctccatccagtctattaattgttgccgggaagctaga<br>gtaagtagttcgccagttaatagtttgcgcaacgttgttgccattgctacaggcatcgtggtgtcacgctcgtcgtttggtatggcttcattcagctccggttcccaacgatcaaggcgagttacatgatcccccat<br>gttgtgcaaaaaagcggttagctccttcggtcctccgatcgttgtcagaagtaagttggccgcagtgttatcactcatggttatggcagcactgcataattctcttactgtcatgccatccgtaagatgcttttctgtg<br>actggtgagtactcaaccaagtcattctgagaatagtgtatgcggcgaccgagttgctcttgcccggcgtcaatacgggataataccgcgccacatagcagaactttaaaagtgctcatcattggaaaacgttc<br>ttcggggcgaaaactctcaaggatcttaccgctgttgagatccagttcgatgtaacccactcgtgcacccaactgatcttcagcatcttttactttcaccagcgtttctgggtgagcaaaaacaggaaggcaaaat

	gccgcaaaaaagggaataagggcgacacggaaatgttgaatactcatactcttcctttttcaatattattgaagcatttatcagggttattgtctcatgagcggatacatatttgaatgtatttagaaaaataaacaaa<br>taggggttccgcgcacatttccccgaaaagtgccacctgacgtcgacggatcgggag
pAAV plasmid<br><br>(gene position described as XXX)	gtcctgcaggcagctgcgcgctcgctcgctcactgaggccgcccgggcaaagcccgggcgtcgggcgacctttggtcgcccggcctcagtgagcgagcgagcgcgcagagagggagtggccaactc<br>catcactaggggttcctgcggccgcacgcgtgtgtctagactgcagagggccctgcgtatgagtgcaagtgggttttaggaccaggatgaggcggggtgggggtgcctacctgacgaccgaccccgacc<br>cactggacaagcacccaacccccattccccaaattgcgcatcccctatcagagagggggaggggaaacaggatgcggcgaggcgcgtgcgcactgccagcttcagcaccgcggacagtgccttcgccc<br>ccgcctggcggcgcgcgccaccgccgcctcagcactgaaggcgcgctgacgtcactcgccggtcccccgcaaactccccttcccggccaccttggtcgcgtccgcgccgccgccggcccagccggac<br>cgcaccacgcgaggcgcgagatagggggcacgggcgcgaccatctgcgctgcggcgccggcgactcagcgctgcctcagtctgcggtgggcagcggaggagtcgtgtcgtgcctgagagcgcag<br>tcgagaaggtaccggatccgccaccXXXXXXXXXXXXXXXXXXtgagaattcgatatcaagcttatcgataatcaacctctggattacaaaatttgtgaaagattgactggtattcttaactat<br>gttgctccttttacgctatgtggatacgctgctttaatgcctttgtatcatgctattgcttcccgtatggctttcattttctcctccttgtataaatcctggttgctgtctctttatgaggagttgtggcccgttgtcaggcaa<br>cgtggcgtggtgtgcactgtgtttgctgacgcaacccccactggttggggcattgccaccacctgtcagctcctttccgggactttcgctttccccctccctattgccacggcggaactcatcgccgcctgcctt<br>gcccgctgctggacaggggctcggctgttgggcactgacaattccgtggtgttgtcggggaaatcatcgtcctttccttggctgctcgcctgtgttgccacctggattctgcgcgggacgtccttctgctacgtc<br>ccttcggccctcaatccagcggaccttccttcccgcggcctgctgccggctctgcggcctcttccgcgtcttcgccttcgccctcagacgagtcggatctccctttgggccgcctccccgcatcgataccgag<br>cgctgctcgagagatctacgggtggcatccctgtgacccctccccagtgcctctcctggccctggaagttgccactccagtgcccaccagccttgtcctaataaaattaagttgcatcattttgtctgactaggtg<br>tccttctataatattatggggtggaggggggtggtatggagcaaggggcaagttgggaagacaacctgtagggcctgcggggtctattgggaaccaagctggagtgcagtggcacaatcttggctcactgc<br>aatctccgcctcctgggttcaagcgattctcctgcctcagcctcccgagttgttgggattccaggcatgcatgaccaggctcagctaatttttgtttttttggtagagacggggtttcaccatattggccaggctggt<br>ctccaactcctaatctcaggtgatctacccaccttggcctcccaaattgctgggattacaggcgtgaaccactgctcccttccctgtccttctgattttgtaggtaaccacgtgcggaccgagcggccgcaggaa<br>cccctagtgatggagttggccactccctctctgcgcgctcgctcgctcactgaggccgggcgaccaaaggtcgcccgacgcccgggctttgcccgggcggcctcagtgagcgagcgagcgcgcagctg<br>cctgcaggggcgcctgatgcggtattttctccttacgcatctgtgcggtatttcacaccgcatacgtcaaagcaaccatagtacgcgccctgtagcggcgcattaagcgcggcgggtgtggtggttacgcgca<br>gcgtgaccgctacacttgccagcgccctagcgcccgctcctttcgctttcttcccttcctttctcgccacgttcgccggctttccccgtcaagctctaaatcgggggctccctttagggttccgatttagtgctttac<br>ggcacctcgaccccaaaaaacttgatttgggtgatggttcacgtagtgggccatcgccctgatagacggtttttcgccctttgacgttggagtccacgttctttaatagtggactcttgttccaaactggaacaaca<br>ctcaaccctatctcgggctattcttttgatttataagggattttgccgatttcggcctattggttaaaaaatgagctgatttaacaaaaatttaacgcgaattttaacaaaatattaacgtttacaattttatggtgcactct<br>cagtacaatctgctctgatgccgcatagttaagccagccccgacacccgccaacacccgctgacgcgccctgacgggcttgtctgctcccggcatccgcttacagacaagctgtgaccgtctccgggagct<br>gcatgtgtcagaggttttcaccgtcatcaccgaaacgcgcgagacgaaagggcctcgtgatacgcctatttttataggttaatgtcatgataataatggtttcttagacgtcaggtggcacttttcggggaaatgt<br>gcgcggaacccctatttgtttatttttctaaatacattcaaatatgtatccgctcatgagacaataaccctgataaatgcttcaataatattgaaaaaggaagagtatgagtattcaacatttccgtgtcgcccttattcc<br>cttttttgcggcattttgccttcctgtttttgctcacccagaaacgctggtgaaagtaaaagatgctgaagatcagttgggtgcacgagtgggttacatcgaactggatctcaacagcggtaagatccttgagagtt<br>ttcgccccgaagaacgttttccaatgatgagcacttttaaagttctgctatgtggcgcggtattatcccgtattgacgccgggcaagagcaactcggtcgccgcatacactattctcagaatgacttggttgagta<br>ctcaccagtcacagaaaagcatcttacggatggcatgacagtaagagaattatgcagtgctgccataaccatgagtgataacactgcggccaacttacttctgacaacgatcggaggaccgaaggagctaac<br>cgcttttttgcacaacatgggggatcatgtaactcgccttgatcgttgggaaccggagctgaatgaagccataccaaacgacgagcgtgacaccacgatgcctgtagcaatggcaacaacgttgcgcaaact<br>attaactggcgaactacttactctagcttcccggcaacaattaatagactggatggaggcggataaagttgcaggaccacttctgcgctcggcccttccggctggctggtttattgctgataaatctggagccgg<br>tgagcgtgggtctcgcggtatcattgcagcactggggccagatggtaagccctcccgtatcgtagttatctacacgacggggagtcaggcaactatggatgaacgaaatagacagatcgctgagataggtg<br>cctcactgattaagcattggtaactgtcagaccaagtttactcatatatactttagattgatttaaaacttcatttttaatttaaaaggatctaggtgaagatcctttttgataatctcatgaccaaaatcccttaacgtgag<br>ttttcgttccactgagcgtcagaccccgtagaaaagatcaaaggatcttcttgagatcctttttttctgcgcgtaatctgctgcttgcaaacaaaaaaaccaccgctaccagcggtggtttgtttgccggatcaaga<br>gctaccaactctttttccgaaggtaactggcttcagcagagcgcagataccaaatactgtccttctagtgtagccgtagttaggccaccacttcaagaactctgtagcaccgcctacatacctcgctctgctaatc<br>ctgttaccagtggctgctgccagtggcgataagtcgtgtcttaccgggttggactcaagacgatagttaccggataaggcgcagcggtcgggctgaacggggggttcgtgcacacagcccagcttggagc<br>gaacgacctacaccgaactgagatacctacagcgtgagctatgagaaagcgccacgcttcccgaagggagaaaggcggacaggtatccggtaagcggcagggtcggaacaggagagcgcacgaggg<br>agcttccagggggaaacgcctggtatctttatagtcctgtcgggtttcgccacctctgacttgagcgtcgatttttgtgatgctcgtcaggggggcggagcctatggaaaaacgccagcaacgcggcctttttac<br>ggttcctggccttttgctggccttttgctcacat

Supplementary Table 3. List of protein genes used in this study.

OLPVR1_His8 codon optimized for *E. Coli* expression	ATGGACAACATCATCATGACCGCCTATATTAGCATTTTCGTGCAGATTATTACCGCCATCATTAGCGTTTATGGTCTGTTTATT<br>CCGCTGAACTTCAAAGATATTATCCTGCGCGAAATTCTGATCCTGGAACTGATTGTTCAGATCATCGAGTTCATCTTCTATAT<br>CTGGCTGATTATTACGCTGCAGAGCATCAATGAAGATATTACCTATGTGCGCTATTTCGATTGGGTTCTGACCACACCGGTTA<br>TGCTGCTGACCACCGTTTATTTCTTTGAGTATATGAATAGCGACGACGGCATCCGCAAAAAAGAAATTAATGATCGCGATTA<br>TGTGTACCTGTTCTACATTTGTCTGAGCAACTTTTTCATGCTGCTTATTGGTTATCTGGGTGAAACCAAGCAGATCAATAAAA<br>TGCTGACCCTGTTTGGTGGTAGCTTTTTTCTGTTTCTGACCTTCTATCTGCTGTACGTGAAATACACCAAAGAAAACTGGATG<br>AACTACATCGTGTTCTATTTCATGTTCCTGGTGTGGTTTCTGTATGGCTTTGCATTTATGTTTCCGTTTAGCATCAAGAACCAG<br>ATGTATAACATTCTGGACATCGTGAGCAAAAACATCTACAGCATCTTTATCTTCATCGTGATTCTGAACCAGAGCTACAAACT<br>GCTCgagagtggtctggtgccgcgcggcagcggtcaccaccatcaccaccatcaccactaa
O1O2(OLPVR1_9 mutant)_His8 codon optimized	ATGGACAACATCATCATGACCGCCTATATTAGCATTTTCGTGCAGATTATTACCGCCATCATTAGCGTTTATGGTCTGTTTATT<br>CCGCTGAACTTCAAAGATATTATCCTGCGCGAAATTCTGATCCTGGAACTGATTGTTCAGATCATCGAGTTCATCTTCTATAT<br>CTGGCTGATTATTACGCTGCAGAGCATCAATGAAGATATTACCTATGTGCGCTATTTCGATTGGGTTCTGACCACACCGGTTA<br>TGCTGCTGACCACCGTTTATTTCTTTGAGTATATGAATAGCGACGACGGCATCCGCAAAAAAGAAATTAATGATCGCGATTA<br>TGTGTACCTGTTCTACATTTGTCTGAGCAACTTTTTCATGCTGCTTATTGGTTATCTGGGTGAAACCAAGCAGATCAATAAAA<br>TGCTGACCCTGTTTGGTGGTAGCTTTTTTCTGTTTCTGACCTTCTATCTGCTGTACGTGAAATACACCAAAGAAAACTGGATG<br>AACTACctgGTGTTCggcgcgATGTTCCTGctgTGGgcgCTGTATGGCTTTGCATTTATGTTTCCGTTTAGCATCAAGAACCAGATGT

for *E. Coli* expression	ATAACATTCTGGACATCtttAGCAAAAACttcgtgAGCATCTTTtttTTCATCGTGATTCTGAACCAGAGCTACAAACTGCTCgagagtggt<br>ctggtgccgcgcggcagcggtcaccaccatcaccaccatcaccactaa
BRIL_VirChR1_His8 codon optimized for *E. Coli* expression	ATGcgtaaaagcctgttagctattcttgcagtctcctcgttggtattcagttctgcgtcgtttgccgctgatcttgaagacaattgggaaaccctcaacgacaatttaaaagtgatcgaaaaagcggataacgcg<br>gcgcaagtcaaagacgcgttaacgaagatgcgcgccgcagcgctggatgcgcaaaaagcaacgccgccgaagctcgaagataaatcaccggacagcccggaaatgaaagatttccgccacggtttcga<br>cattctggtcggtcagattgacgacgcgctgaagctggcaaatgaaggtaaagtaaaagaagcgcaggctgctgcagagcaactgaaaacgacccgcaacgcctatattcagaagtatctggtGacgctg<br>gtgccgcgcggcagccaggcgggtaccAAGGATAAAGAACTGCTGCTGCTGACCGTGAAAATTAGCCTGGTTGTTCAGATTATTAGCGGTA<br>TTGTTAGCAGCTTTGGCATCTTTATCAAACTGGCACCGAAAGATTATATCCTGCGCGATATTCTGATTATCGAAACCGTTGTG<br>CAGATTGTGGAAGCCATCTTTTATGTGTATATCTACCTGAGCCTGGAAAGCCTGGATAACAATGTTATTACCAGCCGTCGTTA<br>TTTCGATTGGGTTATTACAACCCCGATTATGCTGATTAGCACCATTCTGTTTATGCGCTATAACATCCGCATCCTGTATGACA<br>AAAGCAAGAACAAACAGAACCGTCAGAATAAGCCGAAAAGCAATCTGACCACCTATAACGTGATCAAGAACAACAAAGAC<br>ACCATCATCAAAATCGTGATCGGCAACTTTATCATGCTGTGCTTTGGTTATCTGGGCGAAGTTGATATTCTGTCCAAAACCAT<br>TAGCATTCCGATCGGCTTTATCTTCTTCTTCTATGTGTTTTACCTGATCTGGCGTGAATTTGGTAGCTATACCCAGCAGAACCA<br>GATTCTGTTTTGGGTGCTGTTTATTGTTTGGGGTCTGTATGGTGTTAGCGCATGTCTGCCGGTGCTGCAGAAAAACATTTGCT<br>ATAATATCCTGGACATCATCAGCAAGAACTTCTATGGCCTGTATATCTTCTACTACATTTATCAGCATCGTCTGGTGGTGCTCg<br>agagtggtctggtgccgcgcggcagcggtcaccaccatcaccaccatcaccactaa
HF_VirChR1_TS_P2A_Katushka codon optimized for *Homo Sapiens*	ATGAAGACGATCATCGCCCTGAGCTACATCTTCTGCCTGGTATTCGCCGACTACAAGGACGATGATGACGCCAAAggtaccAAG<br>GACAAAGAACTGCTGCTGCTGACCGTGAAGATCAGCCTGGTGGTGCAGATCATCAGCGGCATCGTGTCCAGCTTCGGCATCT<br>TCATCAAGCTGGCCCCTAAGGACTACATCCTGCGGGACATCCTGATCATCGAGACAGTGGTCCAGATCGTGGAAGCCATCTT<br>CTACGTGTACATCTACCTGAGCCTGGAAAGCCTGGACAACAACGTGATCACCAGCCGGCGGTACTTCGACTGGGTCATCACC<br>ACACCTATCATGCTGATCTCCACCATCCTGTTCATGCGGTACAACATCCGCATCCTGTACGACAAGAGCAAGAACAAGCAGA<br>ACCGGCAGAACAAGCCCAAGAGCAACCTGACCACCTACAACGTCATCAAGAACAACAAGGACACCATCATCAAGATCGTGA<br>TCGGGAACTTCATCATGCTGTGCTTCGGCTACCTGGGCGAAGTGGATATCCTGAGCAAGACCATCAGCATCCCTATCGGCTT<br>CATCTTCTTTTTTTACGTGTTCTACCTGATTTGGCGCGAGTTCGGCAGCTACACCCAGCAGAACCAGATCCTGTTCTGGGTGC<br>TGTTCATCGTGTGGGGCCTGTATGGCGTGTCAGCCTGTCTGCCTGTGCTGCAGAAGAACATCTGCTACAATATCCTGGACATC<br>ATCAGCAAGAACTTCTACGGCCTGTACATCTTTTACTACATCTATCAGCATCGGCTGGTGGTCgcggccgcCAAGAGCAGGATCA<br>CCAGCGAGGGCGAGTACATCCCCCTGGACCAGATCGACATCAACGTGAccgcggGCGGCAGCGGCGCCACTAACTTCAGCCTC<br>CTCAAGCAGGCCGGCGACGTGGAGGAGAACCCCGGCCCCggcaccATGGTGGGTGAGGATAGCGTGCTGATCACCGAGAACAT<br>GCACATGAAACTGTACATGGAGGGCACCGTGAACGACCACCACTTCAAGTGCACATCCGAGGGCGAAGGCAAGCCCTACGA<br>GGGCACCCAGACCATGAAGATCAAGGTGGTCGAGGGCGGCCCTCTCCCCTTCGCCTTCGACATCCTGGCTACCAGCTTCATG<br>TACGGCAGCAAAACCTTTATCAACCACACCCAGGGCATCCCCGACTTCTTTAAGCAGTCCTTCCCTGAGGGCTTCACATGGG<br>AGAGGATCACCACATACGAAGACGGGGGCGTGCTGACCGCTACCCAGGACACCAGCCTCCAGAACGGCTGCCTCATCTACA<br>ACGTCAAGATCAACGGGGTGAACTTCCCATCCAACGGCCCTGTGATGCAGAAGAAAACACTCGGCTGGGAGGCCAGCACCG<br>AGATGCTGTACCCCGCTGACAGCGGCCTGAGAGGCCATGCCCAGATGGCCCTGAAGCTCGTGGGCGGGGGCTACCTGCACT<br>GCTCCCTCAAGACCACATACAGATCCAAGAAACCCGCTAAGAACCTCAAGATGCCCGGCTTCTACTTCGTGGACAGGAGACT<br>GGAAAGAATCAAGGAGGCCGACAAAGAGACCTACGTCGAGCAGCACGAGATGGCTGTGGCCAGGTACTGCGACCTGCCTA<br>GCAAACTGGGGCACAGCTGA
VirChR1_TS_P2A_Katushka codon optimized for *Homo Sapiens*	ATGAAGGACAAAGAACTGCTGCTGCTGACCGTGAAGATCAGCCTGGTGGTGCAGATCATCAGCGGCATCGTGTCCAGCTTCG<br>GCATCTTCATCAAGCTGGCCCCTAAGGACTACATCCTGCGGGACATCCTGATCATCGAGACAGTGGTCCAGATCGTGGAAGC<br>CATCTTCTACGTGTACATCTACCTGAGCCTGGAAAGCCTGGACAACAACGTGATCACCAGCCGGCGGTACTTCGACTGGGTC<br>ATCACCACACCTATCATGCTGATCTCCACCATCCTGTTCATGCGGTACAACATCCGCATCCTGTACGACAAGAGCAAGAACA<br>AGCAGAACCGGCAGAACAAGCCCAAGAGCAACCTGACCACCTACAACGTCATCAAGAACAACAAGGACACCATCATCAAG<br>ATCGTGATCGGGAACTTCATCATGCTGTGCTTCGGCTACCTGGGCGAAGTGGATATCCTGAGCAAGACCATCAGCATCCCTA<br>TCGGCTTCATCTTCTTTTTTTACGTGTTCTACCTGATTTGGCGCGAGTTCGGCAGCTACACCCAGCAGAACCAGATCCTGTTCT<br>GGGTGCTGTTCATCGTGTGGGGCCTGTATGGCGTGTCAGCCTGTCTGCCTGTGCTGCAGAAGAACATCTGCTACAATATCCTG<br>GACATCATCAGCAAGAACTTCTACGGCCTGTACATCTTTTACTACATCTATCAGCATCGGCTGGTGGTCgcggccgcCAAGAGCA<br>GGATCACCAGCGAGGGCGAGTACATCCCCCTGGACCAGATCGACATCAACGTGAccgcggGCGGCAGCGGCGCCACTAACTTC<br>AGCCTCCTCAAGCAGGCCGGCGACGTGGAGGAGAACCCCGGCCCCggcaccATGGTGGGTGAGGATAGCGTGCTGATCACCGA<br>GAACATGCACATGAAACTGTACATGGAGGGCACCGTGAACGACCACCACTTCAAGTGCACATCCGAGGGCGAAGGCAAGCC<br>CTACGAGGGCACCCAGACCATGAAGATCAAGGTGGTCGAGGGCGGCCCTCTCCCCTTCGCCTTCGACATCCTGGCTACCAGC<br>TTCATGTACGGCAGCAAAACCTTTATCAACCACACCCAGGGCATCCCCGACTTCTTTAAGCAGTCCTTCCCTGAGGGCTTCAC<br>ATGGGAGAGGATCACCACATACGAAGACGGGGGCGTGCTGACCGCTACCCAGGACACCAGCCTCCAGAACGGCTGCCTCAT<br>CTACAACGTCAAGATCAACGGGGTGAACTTCCCATCCAACGGCCCTGTGATGCAGAAGAAAACACTCGGCTGGGAGGCCAG<br>CACCGAGATGCTGTACCCCGCTGACAGCGGCCTGAGAGGCCATGCCCAGATGGCCCTGAAGCTCGTGGGCGGGGGCTACCT<br>GCACTGCTCCCTCAAGACCACATACAGATCCAAGAAACCCGCTAAGAACCTCAAGATGCCCGGCTTCTACTTCGTGGACAGG<br>AGACTGGAAAGAATCAAGGAGGCCGACAAAGAGACCTACGTCGAGCAGCACGAGATGGCTGTGGCCAGGTACTGCGACCT<br>GCCTAGCAAACTGGGGCACAGCTGA

VirChR1_TS_YFP codon optimized for *Homo Sapiens*	ATGAAGGACAAAGAACTGCTGCTGCTGACCGTGAAGATCAGCCTGGTGGTGCAGATCATCAGCGGCATCGTGTCCAGCTTCG<br>GCATCTTCATCAAGCTGGCCCCTAAGGACTACATCCTGCGGGACATCCTGATCATCGAGACAGTGGTCCAGATCGTGGAAGC<br>CATCTTCTACGTGTACATCTACCTGAGCCTGGAAAGCCTGGACAACAACGTGATCACCAGCCGGCGGTACTTCGACTGGGTC<br>ATCACCACACCTATCATGCTGATCTCCACCATCCTGTTCATGCGGTACAACATCCGCATCCTGTACGACAAGAGCAAGAACA<br>AGCAGAACCGGCAGAACAAGCCCAAGAGCAACCTGACCACCTACAACGTCATCAAGAACAACAAGGACACCATCATCAAG<br>ATCGTGATCGGGAACTTCATCATGCTGTGCTTCGGCTACCTGGGCGAAGTGGATATCCTGAGCAAGACCATCAGCATCCCTA<br>TCGGCTTCATCTTCTTTTTTACGTGTTCTACCTGATTTGGCGCGAGTTCGGCAGCTACACCCAGCAGAACCAGATCCTGTTCT<br>GGGTGCTGTTCATCGTGTGGGGCCTGTATGGCGTGTCAGCCTGTCTGCCTGTGCTGCAGAAGAACATCTGCTACAATATCCTG<br>GACATCATCAGCAAGAACTTCTACGGCCTGTACATCTTTTACTACATCTATCAGCATCGGCTGGTGGTCgcggccgcCAAGAGCA<br>GGATCACCAGCGAGGGCGAGTACATCCCCCTGGACCAGATCGACATCAACGTGGTGAGCAAGGGCGAGGAGCTGTTCACCG<br>GGGTGGTGCCCATCCTGGTCGAGCTGGACGGCGACGTAAACGGCCACAAGTTCAGCGTGTCCGGCGAGGGCGAGGGCGATG<br>CCACCTACGGCAAGCTGACCCTGAAGTTCATCTGCACCACCGGCAAGCTGCCCGTGCCCTGGCCCACCCTCGTGACCACCTT<br>CGGCTACGGCCTGCAGTGCTTCGCCCGCTACCCCGACCACATGAAGCAGCACGACTTCTTCAAGTCCGCCATGCCCGAAGGC<br>TACGTCCAGGAGCGCACCATCTTCTTCAAGGACGACGGCAACTACAAGACCCGCGCCGAGGTGAAGTTCGAGGGCGACACC<br>CTGGTGAACCGCATCGAGCTGAAGGGCATCGACTTCAAGGAGGACGGCAACATCCTGGGGCACAAGCTGGAGTACAACTAC<br>AACAGCCACAACGTCTATATCATGGCCGACAAGCAGAAGAACGGCATCAAGGTGAACTTCAAGATCCGCCACAACATCGAG<br>GACGGCAGCGTGCAGCTCGCCGACCACTACCAGCAGAACACCCCCATCGGCGACGGCCCCGTGCTGCTGCCCGACAACCAC<br>TACCTGAGCTACCAGTCCGCCCTGAGCAAAGACCCCAACGAGAAGCGCGATCACATGGTCCTGCTGGAGTTCGTGACCGCCG<br>CCGGGATCACTCTCGGCATGGACGAGCTGTACAAGTTCTGCTACGAGAACGAGGTGTGA
HF_OLPVR1_TS_P2A_Katushka codon optimized for *Homo Sapiens*	ATGAAGACGATCATCGCCCTGAGCTACATCTTCTGCCTGGTATTCGCCGACTACAAGGACGATGATGACGCCAAAggtaccGAC<br>AACATCATCATGACCGCCTACATCAGCATCTTCGTGCAGATCATCACCGCCATCATCTCCGTGTACGGCCTGTTCATCCCTCT<br>GAACTTCAAGGACATCATCCTGCGCGAGATCCTGATCCTGGAACTGATTGTGCAGATTATCGAGTTCATCTTCTACATCTGGC<br>TCATCATCACCCTGCAGAGCATCAACGAGGACATCACCTACGTGCGGTACTTCGACTGGGTGCTGACCACACCTGTGATGCT<br>GCTGACAACCGTGTACTTCTTCGAGTACATGAACAGCGACGACGGCATCCGGAAGAAAGAGATCAACGACCGGGACTACGT<br>GTACCTGTTCTATATCTGCCTGAGCAACTTCTTCATGCTGCTCATCGGCTACCTGGGCGAGACAAAGCAGATCAACAAAATG<br>CTGACCCTGTTCGGCGGCAGCTTCTTCCTGTTCCTGACCTTCTACCTGCTGTACGTGAAGTACACCAAAGAAAACTGGATGAA<br>CTACATCGTGTTCTACTTTATGTTCCTCGTGTGGTTCCTGTACGGCTTCGCCTTCATGTTCCCATTCAGCATCAAGAACCAGAT<br>GTACAACATCCTGGACATCGTGTCCAAGAACATCTACTCCATCTTCATCTTTATCGTGATCCTGAACCAGAGCTACAAGCTGC<br>TGgcggccgcCAAGAGCAGGATCACCAGCGAGGGCGAGTACATCCCCCTGGACCAGATCGACATCAACGTGAccgcggGCGGCAG<br>CGGCGCCACTAACTTCAGCCTCCTCAAGCAGGCCGGCGACGTGGAGGAGAACCCCGGCCCCggcaccATGGTGGGTGAGGATA<br>GCGTGCTGATCACCGAGAACATGCACATGAAACTGTACATGGAGGGCACCGTGAACGACCACCACTTCAAGTGCACATCCG<br>AGGGCGAAGGCAAGCCCTACGAGGGCACCCAGACCATGAAGATCAAGGTGGTCGAGGGCGGCCCTCTCCCCTTCGCCTTCG<br>ACATCCTGGCTACCAGCTTCATGTACGGCAGCAAAACCTTTATCAACCACACCCAGGGCATCCCCGACTTCTTTAAGCAGTC<br>CTTCCCTGAGGGCTTCACATGGGAGAGGATCACCACATACGAAGACGGGGGCGTGCTGACCGCTACCCAGGACACCAGCCT<br>CCAGAACGGCTGCCTCATCTACAACGTCAAGATCAACGGGGTGAACTTCCCATCCAACGGCCCTGTGATGCAGAAGAAAAC<br>ACTCGGCTGGGAGGCCAGCACCGAGATGCTGTACCCCGCTGACAGCGGCCTGAGAGGCCATGCCCAGATGGCCCTGAAGCT<br>CGTGGGCGGGGGCTACCTGCACTGCTCCCTCAAGACCACATACAGATCCAAGAAACCCGCTAAGAACCTCAAGATGCCCGG<br>CTTCTACTTCGTGGACAGGAGACTGGAAAGAATCAAGGAGGCCGACAAAGAGACCTACGTCGAGCAGCACGAGATGGCTGT<br>GGCCAGGTACTGCGACCTGCCTAGCAAACTGGGGCACAGCTGA
OLPVR1_TS_YFP codon optimized for *Homo Sapiens*	ATGGACAACATCATCATGACCGCCTACATCAGCATCTTCGTGCAGATCATCACCGCCATCATCTCCGTGTACGGCCTGTTCAT<br>CCCTCTGAACTTCAAGGACATCATCCTGCGCGAGATCCTGATCCTGGAACTGATTGTGCAGATTATCGAGTTCATCTTCTACA<br>TCTGGCTCATCATCACCCTGCAGAGCATCAACGAGGACATCACCTACGTGCGGTACTTCGACTGGGTGCTGACCACACCTGT<br>GATGCTGCTGACAACCGTGTACTTCTTCGAGTACATGAACAGCGACGACGGCATCCGGAAGAAAGAGATCAACGACCGGGA<br>CTACGTGTACCTGTTCTATATCTGCCTGAGCAACTTCTTCATGCTGCTCATCGGCTACCTGGGCGAGACAAAGCAGATCAACA<br>AAATGCTGACCCTGTTCGGCGGCAGCTTCTTCCTGTTCCTGACCTTCTACCTGCTGTACGTGAAGTACACCAAAGAAAACTGG<br>ATGAACTACATCGTGTTCTACTTTATGTTCCTCGTGTGGTTCCTGTACGGCTTCGCCTTCATGTTCCCATTCAGCATCAAGAAC<br>CAGATGTACAACATCCTGGACATCGTGTCCAAGAACATCTACTCCATCTTCATCTTTATCGTGATCCTGAACCAGAGCTACAA<br>GCTGCTGgcggccgcCAAGAGCAGGATCACCAGCGAGGGCGAGTACATCCCCCTGGACCAGATCGACATCAACGTGGTGAGCA<br>AGGGCGAGGAGCTGTTCACCGGGGTGGTGCCCATCCTGGTCGAGCTGGACGGCGACGTAAACGGCCACAAGTTCAGCGTGT<br>CCGGCGAGGGCGAGGGCGATGCCACCTACGGCAAGCTGACCCTGAAGTTCATCTGCACCACCGGCAAGCTGCCCGTGCCCT<br>GGCCCACCCTCGTGACCACCTTCGGCTACGGCCTGCAGTGCTTCGCCCGCTACCCCGACCACATGAAGCAGCACGACTTCTT<br>CAAGTCCGCCATGCCCGAAGGCTACGTCCAGGAGCGCACCATCTTCTTCAAGGACGACGGCAACTACAAGACCCGCGCCGA<br>GGTGAAGTTCGAGGGCGACACCCTGGTGAACCGCATCGAGCTGAAGGGCATCGACTTCAAGGAGGACGGCAACATCCTGGG<br>GCACAAGCTGGAGTACAACTACAACAGCCACAACGTCTATATCATGGCCGACAAGCAGAAGAACGGCATCAAGGTGAACTT<br>CAAGATCCGCCACAACATCGAGGACGGCAGCGTGCAGCTCGCCGACCACTACCAGCAGAACACCCCCATCGGCGACGGCCC<br>CGTGCTGCTGCCCGACAACCACTACCTGAGCTACCAGTCCGCCCTGAGCAAAGACCCCAACGAGAAGCGCGATCACATGGT<br>CCTGCTGGAGTTCGTGACCGCCGCCGGGATCACTCTCGGCATGGACGAGCTGTACAAGTTCTGCTACGAGAACGAGGTGTGA

HF_DTSRh_TS_P2A_Katushka codon optimized for *Homo Sapiens*	ATGAAGACGATCATCGCCCTGAGCTACATCTTCTGCCTGGTATTCGCCGACTACAAGGACGATGATGACGCCAAAggtaccGAA<br>AACAGCTTCATCGTGAAGAATACGATGATCTTCAGCTTCCTGATCCAGATCATCACCCTGATCATCGGCATCTTCGCCCAGTT<br>CATCAAGGTGCCCCGGCACAAGTACATCCTGAAGGACGCTCTGCTGCTGGAAAACATCGTGCAGTTCATCGAGGCCATCTTC<br>TACCTGTGGTTCATCTACTTCTACAAAGAGAACGTCGACAAGATCGATATCGCCAAGTACCGCTACTACGACTGGTTCCTGA<br>CCACACCTACCATGATCCTGAGCGTGATCATCTATTTCCACTACAACAACAGCAGCAAGAAAATCTACTACAACATGATCAC<br>CTTTTTCAAGCAAGATTTCCGGAAGATCCTGGAACTGTGGTTCTACAACTTCAACATGCTCATCATCGGCTACCTGCAAGAGA<br>TCAACATCATCAGCATCCTGCTGTCTACCCTGATCGGCTTCTACTTCTTCGGCCTGCTGTTCTACAAGATGTTCAAGTACTACG<br>TGGTGCAGAACAAGAAGAACTACCTCCTGTTCTTCCTGATGTTCTTCATCTGGGGCCTGTACGGAATCGCCGCTCTGTTCAAC<br>TACAAGTTCAAGAACGCCTTCTACAACATCCTGGATATCTTCAGCAAGAATTTCTTCGGGCTGTTCCTGGCCTACCTGGTGTT<br>TACCGGAgcggccgcCAAGAGCAGGATCACCAGCGAGGGCGAGTACATCCCCCTGGACCAGATCGACATCAACGTGAccgcggGC<br>GGCAGCGGCGCCACTAACTTCAGCCTCCTCAAGCAGGCCGGCGACGTGGAGGAGAACCCCGGCCCCggcaccATGGTGGGTGA<br>GGATAGCGTGCTGATCACCGAGAACATGCACATGAAACTGTACATGGAGGGCACCGTGAACGACCACCACTTCAAGTGCAC<br>ATCCGAGGGCGAAGGCAAGCCCTACGAGGGCACCCAGACCATGAAGATCAAGGTGGTCGAGGGCGGCCCTCTCCCCTTCGC<br>CTTCGACATCCTGGCTACCAGCTTCATGTACGGCAGCAAAACCTTTATCAACCACACCCAGGGCATCCCCGACTTCTTTAAGC<br>AGTCCTTCCCTGAGGGCTTCACATGGGAGAGGATCACCACATACGAAGACGGGGGCGTGCTGACCGCTACCCAGGACACCA<br>GCCTCCAGAACGGCTGCCTCATCTACAACGTCAAGATCAACGGGGTGAACTTCCCATCCAACGGCCCTGTGATGCAGAAGAA<br>AACACTCGGCTGGGAGGCCAGCACCGAGATGCTGTACCCCGCTGACAGCGGCCTGAGAGGCCATGCCCAGATGGCCCTGAA<br>GCTCGTGGGCGGGGGCTACCTGCACTGCTCCCTCAAGACCACATACAGATCCAAGAAACCCGCTAAGAACCTCAAGATGCC<br>CGGCTTCTACTTCGTGGACAGGAGACTGGAAAGAATCAAGGAGGCCGACAAAGAGACCTACGTCGAGCAGCACGAGATGGC<br>TGTGGCCAGGTACTGCGACCTGCCTAGCAAACTGGGGCACAGCTGA
LR_His8 codon optimized for *Leishmania tarentolae*	ATGATCGTGGACCAGTTCGAGGAGGTGCTGATGAAGACGAGCCAGCTGTTTCCGCTGCCGACAGCAACACAGAGCGCACAG<br>CCAACACATGTGGCGCCAGTGCCAACAGTGCTGCCGGACACACCAATCTACGAGACAGTGGGCGACAGCGGCAGCAAGACA<br>CTGTGGGTCGTGTTCGTGCTGATGCTGATTGCGAGCGCGGCGTTTACAGCGCTGAGCTGGAAGATTCCAGTGAACCGCCGTC<br>TGTACCACGTGATCACCACCATCATCACGCTGACAGCGGCCCTGAGCTACTTCGCAATGGCAACTGGTCATGGCGTGGCGCT<br>GAACAAGATCGTGATTCGCACACAGCACGACCACGTGCCAGACACGTACGAGACTGTGTACCGCCAGGTGTACTACGCGCG<br>CTACATCGACTGGGCGATTACGACACCACTGCTGCTGCTGGATCTGGGCCTTCTTGCAGGCATGAGCGGCGCACACATCTTC<br>ATGGCGATTGTGGCGGACCTGATCATGGTGCTGACTGGCCTGTTTGCGGCGTTTGGCTCTGAGGGCACACCACAGAAGTGGG<br>GCTGGTACACGATCGCCTGCATTGCCTACATCTTCGTCGTGTGGCACCTGGTGCTGAACGGCGGTGCAAACGCACGTGTGAA<br>GGGCGAGAAGCTGCGCTCTTTCTTTGTGGCGATTGGCGCGTACACGCTGATCCTGTGGACAGCGTACCCAATCGTGTGGGGT<br>CTTGCAGACGGCGCACGCAAGATCGGTGTCGACGGCGAGATTATTGCGTACGCGGTGCTGGACGTGCTGGCGAAGGGCGTG<br>TTCGGTGCATGGCTGCTTGTGACACATGCGAACCTTCGCGAGAGCGACGTCGAGCTGAACGGTTTTTGGGCGAACGGCCTTA<br>ACCGCGAGGGCGCAATTCGTATTGGCGAGGACGATGGTGCAGGCACGCACCACCATCACCATCATCACCACCAT

Supplementary Table 4. List of primers used in this study.

_forw_VirChR1_humanized	GCCAAAAAGGACAggtaccAAGAACTGCTGCTG
_rev_VirChR1_humanized	CTGCTCTTGgcggccgcGAC
_forw_OLPVR1_humanized	GCCAAAggtaccGACAACATCATCATGACC
_rev_OLPVR1_humanized	CTCTTGgcggccgcCAGC
_forw_DTSRh_humanized	GCCAAAggtaccGAAAACAGCTTCATCGTG
_rev_DTSRh_humanized	CTCTTGgcggccgcTCCGGTAAACAC

_forw_OLPVR1_ecoli	gatatacatATGGACAACATCATCATGACCGCCTATATTAGC
_rev_OLPVR1_ecoli	accactctcGAGCAGTTTGTAGCTCTGGTTC
_forw_VirChR1_ecoli	gatatacatATGAAGGATAAAGAACTGCTGCTGCTGACC
_rev_VirChR1_ecoli	accactctcGAGCACCACCAGACGATG

Supplementary References

1. Yutin, N. & Koonin, E. V. Proteorhodopsin genes in giant viruses. *Biol. Direct* (2012) doi:10.1186/1745-6150-7-34.
2. Needham, D. M. *et al.* A distinct lineage of giant viruses brings a rhodopsin photosystem to unicellular marine predators. *Proc. Natl. Acad. Sci.* (2019) doi:10.1073/pnas.1907517116.
3. Pushkarev, A. *et al.* A distinct abundant group of microbial rhodopsins discovered. *Nature* (2018) doi:10.1038/s41586-018-0225-9.
4. Kovalev, K. *et al.* High-resolution structural insights into the heliorhodopsin family. *Proc. Natl. Acad. Sci. U. S. A.* (2020) doi:10.1073/pnas.1915888117.
5. Mitchell, A. L. *et al.* InterPro in 2019: improving coverage, classification and access to protein sequence annotations. *Nucleic Acids Res.* (2019) doi:10.1093/nar/gky1100.
6. Okonechnikov, K. *et al.* Unipro UGENE: A unified bioinformatics toolkit. *Bioinformatics* (2012) doi:10.1093/bioinformatics/bts091.
7. Letunic, I. & Bork, P. Interactive tree of life (iTOL) v3: an online tool for the display and annotation of phylogenetic and other trees. *Nucleic Acids Res.* (2016) doi:10.1093/nar/gkw290.

8. Oppermann, J. *et al.* MerMAIDs: a family of metagenomically discovered marine anion-conducting and intensely desensitizing channelrhodopsins. *Nat. Commun.* (2019) doi:10.1038/s41467-019-11322-6.

9. Baek, M., Park, T., Heo, L., Park, C. & Seok, C. GalaxyHomomer: A web server for protein homo-oligomer structure prediction from a monomer sequence or structure. *Nucleic Acids Res.* (2017) doi:10.1093/nar/gkx246.

10. Ran, T. *et al.* Cross-protomer interaction with the photoactive site in oligomeric proteorhodopsin complexes. *Acta Crystallogr. D Biol. Crystallogr.* (2013) doi:10.1107/S0907444913017575.

11. Kim, Y. S. *et al.* Crystal structure of the natural anion-conducting channelrhodopsin GtACR1. *Nature* (2018) doi:10.1038/s41586-018-0511-6.

12. Oda, K. *et al.* Crystal structure of the red light-activated channelrhodopsin Chrimson. *Nat. Commun.* (2018) doi:10.1038/s41467-018-06421-9.

13. Luecke, H., Schobert, B., Richter, H. T., Cartailler, J. P. & Lanyi, J. K. Structure of bacteriorhodopsin at 1.55 Å resolution. *J. Mol. Biol.* **291**, 899–911 (1999).

14. Bratanov, D. *et al.* Unique structure and function of viral rhodopsins. *Nat. Commun.* **10**, 4939 (2019).

15. Kato, H. E. *et al.* Crystal structure of the channelrhodopsin light-gated cation channel. *Nature* (2012) doi:10.1038/nature10870.

16. Ho, B. K. & Gruswitz, F. HOLLOW: Generating accurate representations of channel and interior surfaces in molecular structures. *BMC Struct. Biol.* **8**, (2008).
